# PDGFRα^+^DPP4^+^ Fibroblasts‐Macrophage Crosstalk Induces Orbital Fibrosis in Treatment‐Resistant Thyroid Eye Disease via the GAS6‐AXL Pathway

**DOI:** 10.1002/advs.202511404

**Published:** 2025-09-29

**Authors:** Lu Cheng, Jinwei Cheng, Guiling Liang, Xiaorui Wang, Yanwen Ge, Jiapei Liu, Luwei Cai, Hui Ying, Fenfen Wang, Jing Hu, Yufan Wang, Philipp E. Scherer, Ben Zhou, Mengle Shao, Fang Zhang

**Affiliations:** ^1^ National Clinical Research Center for Eye Diseases Shanghai General Hospital Shanghai Jiao Tong University School of Medicine Shanghai 200080 China; ^2^ Institute of Ophthalmology Department of Ophthalmology Shanghai General Hospital Shanghai Jiao Tong University School of Medicine Shanghai 200080 China; ^3^ Department of Endocrinology and Metabolism Shanghai General Hospital Shanghai Jiao Tong University School of Medicine Shanghai 200080 China; ^4^ Touchstone Diabetes Center Department of Internal Medicine The University of Texas Southwestern Medical Center at Dallas Dallas TX 75390 USA; ^5^ CAS Key Laboratory of Nutrition, Metabolism and Food Safety Shanghai Institute of Nutrition and Health University of Chinese Academy of Sciences Chinese Academy of Sciences Shanghai 200031 China; ^6^ State Key Laboratory of Immune Response and Immunotherapy Shanghai Institute of Materia Medica Chinese Academy of Sciences Shanghai 200031 China

**Keywords:** fibrosis, GAS6‐AXL pathway, macrophage spatial distribution, orbital adipose tissue, thyroid eye disease

## Abstract

Thyroid eye disease (TED), the leading adult orbital disease, is an autoimmune disorder characterized by fibrosis. Effective anti‐fibrotic treatments are scarce, except for orbital decompression surgery involving orbital adipose tissue (OAT) removal, due to high rates of drug resistance following hyperthyroidism treatment and the lack of suitable mouse models. Understanding the mechanisms behind fibrotic remodeling of OAT could aid mouse model development and identify novel therapies. In the present study, stromal vascular fraction cells of OAT from patients with inactive‐stage TED, characterized by pronounced fibrosis, are analyzed at single‐cell resolution. platelet‐derived growth factor receptor (PDGFR)α^+^dipeptidyl peptidase (DPP)4^+^ fibroblasts exhibiting progenitor characteristics and fibrotic potential at the transcriptional level are identified. PDGFRα^+^DPP4^+^ fibroblasts showed the strongest interactions with macrophages, particularly M2 macrophages, which are enriched and topographically localized within the fibrotic area. Moreover, M2 macrophages promoted extracellular matrix production in PDGFRα^+^DPP4^+^ cells via the Growth arrest specific (GAS)6‐AXL Receptor Tyrosine Kinase (AXL) signaling pathway. Using a specific AXL inhibitor or AXL knockdown, fibrosis is substantially reduced in PDGFRα^+^DPP4^+^ fibroblasts in vitro, and in patient cell‐derived orthotopic xenograft models established via GAS6. By identifying pro‐fibrotic intercellular networks in OAT, these findings establish a rapid and repeatable mouse model of TED fibrosis and propose the GAS6‐AXL axis as a potential therapeutic target for TED.

## Graphical abstract

This study elucidates macrophage‐induced orbital fibrosis in TED via the GAS6‐AXL pathway, a treatment‐resistant disorder lacking non‐surgical therapies. Using a novel patient cell‐derived orthotopic animal model of orbital fibrosis for TED, we repurpose an existing fibrosis therapy, addressing the absence of stable models and offering a potential treatment avenue.

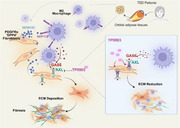

## 1 Introduction

Thyroid eye disease (TED), the leading adult orbital condition (also known as Graves’ orbitopathy or thyroid‐associated orbitopathy), involves hyperthyroidism and ophthalmopathy.^[^ ^1^ ^]^ A significant challenge is the limited efficacy of anti‐thyroid treatments on ophthalmopathy. For instance, radioiodine therapy, a common treatment for hyperthyroidism, has been shown in clinical studies to paradoxically trigger the onset or exacerbation of ophthalmopathy.^[^ ^2^, ^3^ ^]^ This risk is acknowledged in major clinical guidelines, which note that the majority of TED patients develop ocular manifestations while undergoing treatment for hyperthyroidism.^[^ ^4^ ^]^ Furthermore, the thyroid‐stimulating hormone receptor (TSHR) serves as the primary autoantigen and is known to be pivotal in the pathogenesis of TED.^[^ ^5^, ^6^ ^]^ Current TED animal models are mechanistically based on the induction of anti‐TSHR antibodies (TRAbs) in experimental animals. The most common experimental models employ either plasmid electroporation or adenovirus‐mediated transfection of the human TSHR‐A subunit.^[^ ^5^, ^7^ ^]^ Despite their application, these methods suffer from notable drawbacks, including low success rates, prolonged induction periods, and poor reproducibility.^[^ ^8^ ^]^ These limitations may stem from mechanistic differences from the autoimmune reactions that occur in humans.^[^ ^8^, ^9^ ^]^ Consequently, the resistance to anti‐thyroid treatments, coupled with the lack of a stable animal model, hinders the development of effective therapeutic agents for TED.

TED primarily follows a biphasic course: an active stage marked by inflammation and excessive adipogenesis, and an inactive stage characterized by fibrosis.^[^ ^10^, ^11^, ^12^ ^]^ Consequences include permanent eye motility impairment, proptosis, and compressive optic neuropathy.^[^ ^13^, ^14^ ^]^ While several treatments offer partial benefit for active‐stage TED, including glucocorticoids,^[^ ^15^, ^16^ ^]^ immunosuppressive agents,^[^ ^17^ ^]^ orbital radiotherapy,^[^ ^18^ ^]^ and biological agents such as teprotumumab, an insulin‐like growth factor I receptor (IGF‐IR) inhibitor,^[^ ^19^ ^]^ sirolimus,^[^ ^20^ ^]^ tocilizumab,^[^ ^21^ ^]^ batoclimab,^[^ ^22^ ^]^ and atorvastatin,^[^ ^23^ ^]^ effective medical interventions for fibrosis in inactive‐stage TED remain elusive. Although certain antifibrotics, such as nintedanib and pirfenidone, originally developed for pulmonary fibrosis,^[^ ^24^, ^25^ ^]^ have been trialed in fibrotic eye diseases such as fibrotic cataracts,^[^ ^26^, ^27^ ^]^ their efficacy in TED‐related fibrosis has not been established. Currently, the primary treatment is orbital decompression surgery, which involves removing the extra fibrotic orbital adipose tissue (OAT).^[^ ^28^ ^]^ Therefore, developing drug‐based alternatives to surgery remains a challenge despite the urgent need.^[^ ^16^, ^29^ ^]^

Previous studies on the pathogenesis of fibrosis OAT in TED indicated that orbital fibroblasts contribute to fibrosis by depositing excess extracellular matrix (ECM), primarily collagen types I and III, and fibronectin, alongside ECM regulators such as tissue inhibitor of metalloproteinases‐1 (TIMP1) and matrix metalloproteinase‐1 (MMP1), in fibrotic adipose tissue.^[^ ^13^, ^30^ ^]^ THY1^+^ (CD90^+^) fibroblasts are suggested to be the primary cells differentiating into myofibroblasts in TED‐related fibrosis.^[^ ^31^, ^32^ ^]^ As an autoimmune disorder,^[^ ^33^, ^34^ ^]^ the orbital immunity microenvironment also plays a role in TED‐related fibrosis. T cells, as the most studied immune cells in TED, contribute to the disease progression through costimulatory molecules, adhesion molecules, and cytokines.^[^ ^35^ ^]^ T cell‐derived cytokines, such as interferon‐γ (IFN‐γ), interleukin‐17 (IL‐17), and interleukin‐4 (IL‐4), in OAT have been shown to stimulate differentiation of circulating monocyte‐derived fibrocytes or resident fibroblasts into myofibroblasts.^[^ ^35^, ^36^, ^37^ ^]^ Excessive ECM production can be stimulated by transforming growth factor‐β (TGFβ), or through direct cell–cell contact with immune cells infiltrating the inflamed orbit, such as CD40–CD154.^[^ ^38^ ^]^ In addition to the significant research conducted on T cells, recent reports have revealed that there is an elevated presence of platelet‐derived growth factor (PDGF)‐β‐positive monocytes, macrophages, and mast cells within the OAT of patients with TED.^[^ ^13^, ^39^ ^]^ However, there remains a translational gap between identifying putative targets and developing effective therapies for treating fibrosis in TED, largely due to the limited understanding of the functional heterogeneity and interactome in the stromal vascular fraction (SVF) cell lineages of OAT fibrosis.

Single‐cell RNA sequencing (scRNA‐seq) suggests an unbiased and comprehensive view of cell heterogeneity and potential intercellular crosstalk. It has been widely applied to precisely identify cell subsets related to adipocyte differentiation, inflammation, and fibrosis in peripheral adipose tissue.^[^ ^40^, ^41^, ^42^, ^43^, ^44^ ^]^ In contrast to the numerous studies on peripheral adipose depots, only two reports related have focused on scRNA‐seq analysis of OAT.^[^ ^36^, ^45^ ^]^ One study investigated the bioinformative prediction of adipogenesis regulation in the OAT of individuals with active‐stage TED,^[^ ^45^ ^]^ which suggests the involvement of THY1^−^Ras Related Dexamethasone Induced (RASD)1^+^ lipo‐fibroblasts in inflammation and adipogenesis. The other study examined T‐cell regulation in TED with a focus on the Th17 cell subsets.^[^ ^36^ ^]^ However, the progenitor cells responsible for OAT fibrosis and the mechanisms driving their activation in TED remain unclear.

This study addresses the unmet need for non‐surgical therapies in TED fibrosis. We uncovered the pathogenic role of the Growth arrest specific (GAS)6‐AXL Receptor Tyrosine Kinase (AXL) pathway in M2 macrophage‐triggered orbital progenitor fibrosis during inactive‐stage TED by characterizing SVF cell heterogeneity and interactions through unbiased scRNA‐seq, immunofluorescence, fluorescence‐activated cell sorting (FACS), and in vitro co‐culture assays. To validate this mechanism, we established a mechanism‐based patient cell‐derived orthotopic animal model to study orbital fibrosis. Guided by these findings, we repurposed an AXL inhibitor for TED treatment. Collectively, our data position GAS6‐AXL inhibition as a potential breakthrough therapeutic strategy for TED fibrosis.

## 2 Results

### 2.1 Single‐cell Atlas of Non‐Adipocyte Fraction from Human OAT Reveals Fibroblasts as the Fibrotic Effector‐Cells in TED

**Figure 1:**
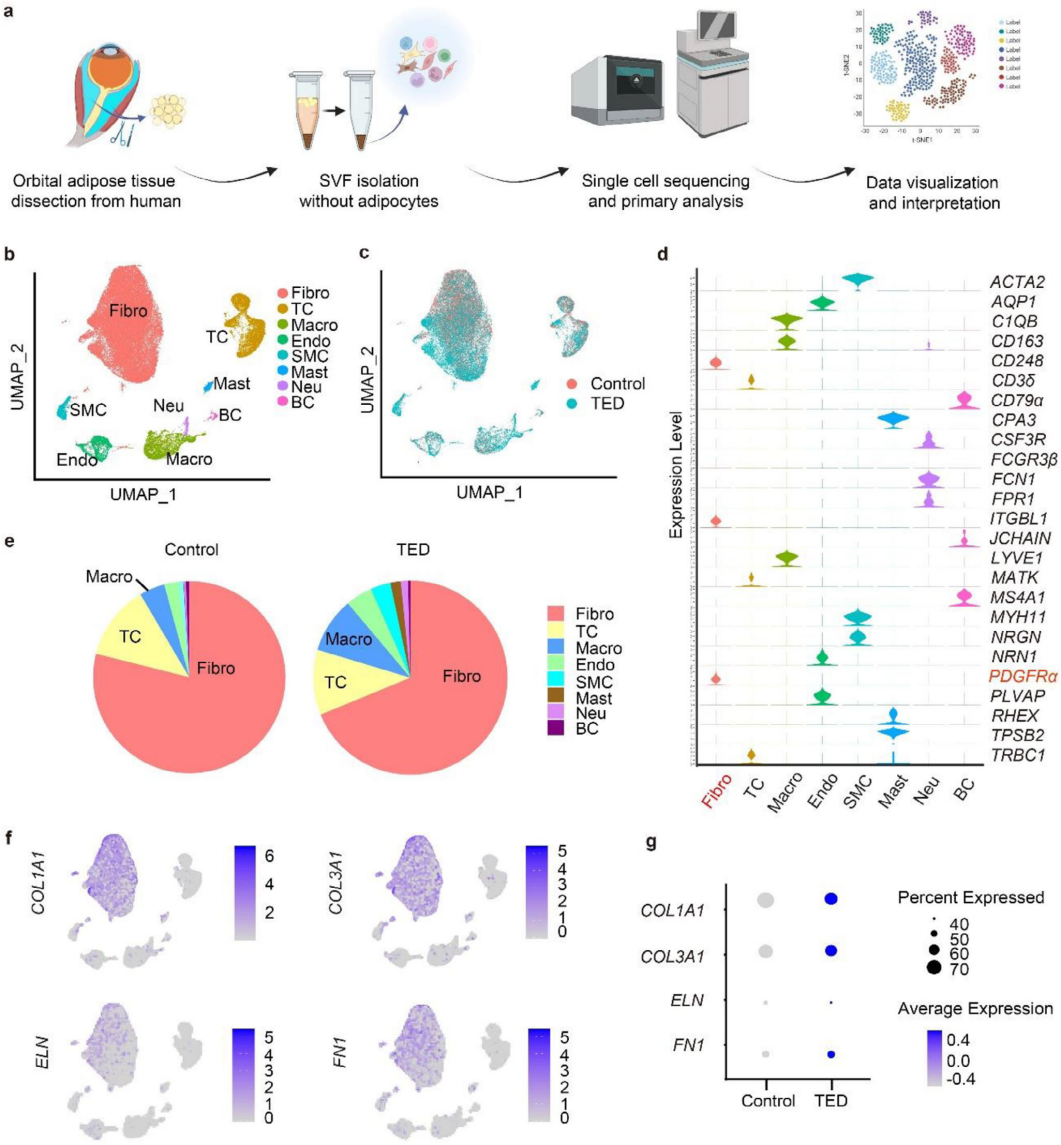
Single‐cell RNA sequencing reveals heterogeneity of orbital adipose‐derived stromal vascular fraction, fibrosis‐effecting cells, and increased macrophage in TED. a) Schematic representation of single‐cell RNA sequencing (scRNA‐seq) for human orbital adipose tissue (OAT)‐derived stromal vascular fraction (SVF). b) UMAP visualization of 58 253 cells from the OAT‐derived SVF in three patients with thyroid eye disease (TED) and two control subjects, revealing eight distinct cell clusters. Each dot represents a single cell. c) UMAP of merged TED (cyan) and control (red) group cells. d) Violin plot of specific marker genes in each cluster. e) Proportions of cell types in TED and control groups. f) Feature plot indicating the distribution and expression of representative fibrosis markers in all the cells. g) Dot plot indicating the expression of representative fibrosis markers in TED and control groups. Dot size represents the proportion of cells expressing the denoted gene. Color indicates the average normalized expression level within the cluster.

To investigate the pathogenesis of OAT fibrosis in TED, we performed scRNA‐seq on the OAT‐derived SVF cells isolated from three patients with inactive‐stage TED undergoing decompression surgery and two patients with orbital fat prolapse without TED as controls (**Figure** **1**a). General patient information is summarized in Table S1. After quality control, we clustered 58 253 OAT‐derived SVF cells (29 712 cells from TED and 28 541 cells from control subjects) according to known lineage marker signatures. The cell annotation results were then visualized by a uniform manifold approximation and projection (UMAP) plot (Figure 1b and Figure S1a, Supporting Information), each containing cells from both control and TED groups (Figure 1c). Subpopulation marker genes from various scRNA‐seq datasets were identified across all lineages and visualized with violin and dot plots (Figure 1d and Figure S1b, Supporting Information). In total, we identified eight cell types in the OAT‐derived SVF, including fibroblasts, T cells, macrophages, endothelial cells, smooth muscle cells, mast cells, neutrophils, and B cells. Among these lineages, fibroblasts accounted for the largest proportion in both TED (68.8%) and control groups (78.9%). T cells ranked the largest population in immune cells, with an average of 11.7% in SVF from OAT. The TED‐derived SVF exhibited number ratios of each lineage distinct from the control samples (Figure 1e and Figure S1c, Supporting Information). Macrophages, which ranked the second largest proportion of immune cells in OAT, exhibited a 2.2‐fold increase in TED (9.2%) compared with the control group (4.2%). Slight decreases were seen in T cells (12.6%–10.8%) and B cells (0.6%–0.4%) of the TED group compared with those of the control group. Increased endothelial cells (2.4%–4.5%), smooth muscle cells (0.7%–3.3%), mast cells (0.1%–1.7%), and neutrophils (0.4%–1.2%) were seen in the TED group compared with those of the control group.

To identify the fibrotic‐effector cells of OAT in TED, we investigated the expression and distribution of fibrotic markers in the eight cell types in OAT. Feature plots indicated that the expression of fibrosis‐associated genes, including collagen type I (*COL1A1*), collagen type III (*COL3A1*), elastin (*ELN*), and fibronectin 1 (*FN1*)^[^ ^46^, ^47^ ^]^ were mainly distributed in cells of the fibroblast lineage (Figure 1f). The expression of each marker in the TED group was significantly higher than that in the control group (Figure 1g). Among the canonical fibroblast signature markers, such as PDGF receptor‐α (PDGFRα),^[^ ^43^ ^]^ PDGFRβ,^[^ ^41^ ^]^ CD248,^[^ ^48^ ^]^ and integrin beta‐like 1 (ITGBL1),^[^ ^49^ ^]^ PDGFRα is commonly expressed in all OAT fibroblasts (Figure S1d Supporting Information). Our scRNA‐seq characterized the non‐adipocyte lineages in OAT at cellular and transcriptional levels and identified fibroblasts as the fibrotic effector‐cells in OAT of TED.

All scRNA‐seq analyses presented in the figure are based on a total of five SVF samples, which include two control and three TED‐associated SVF samples. These SVF samples were derived from OAT obtained from patients. SVF, stromal vascular fraction; Fibro, fibroblasts; TC, T cells; Macro, macrophages; Endo, endothelial cells; SMC, smooth muscle cells; Mast, mast cells; Neu, neutrophils; BC, B cells.

### 2.2 Re‐Clustering Analysis of Orbital Fibroblast Heterogeneity and Fibrotic Progenitors

**Figure 2:**
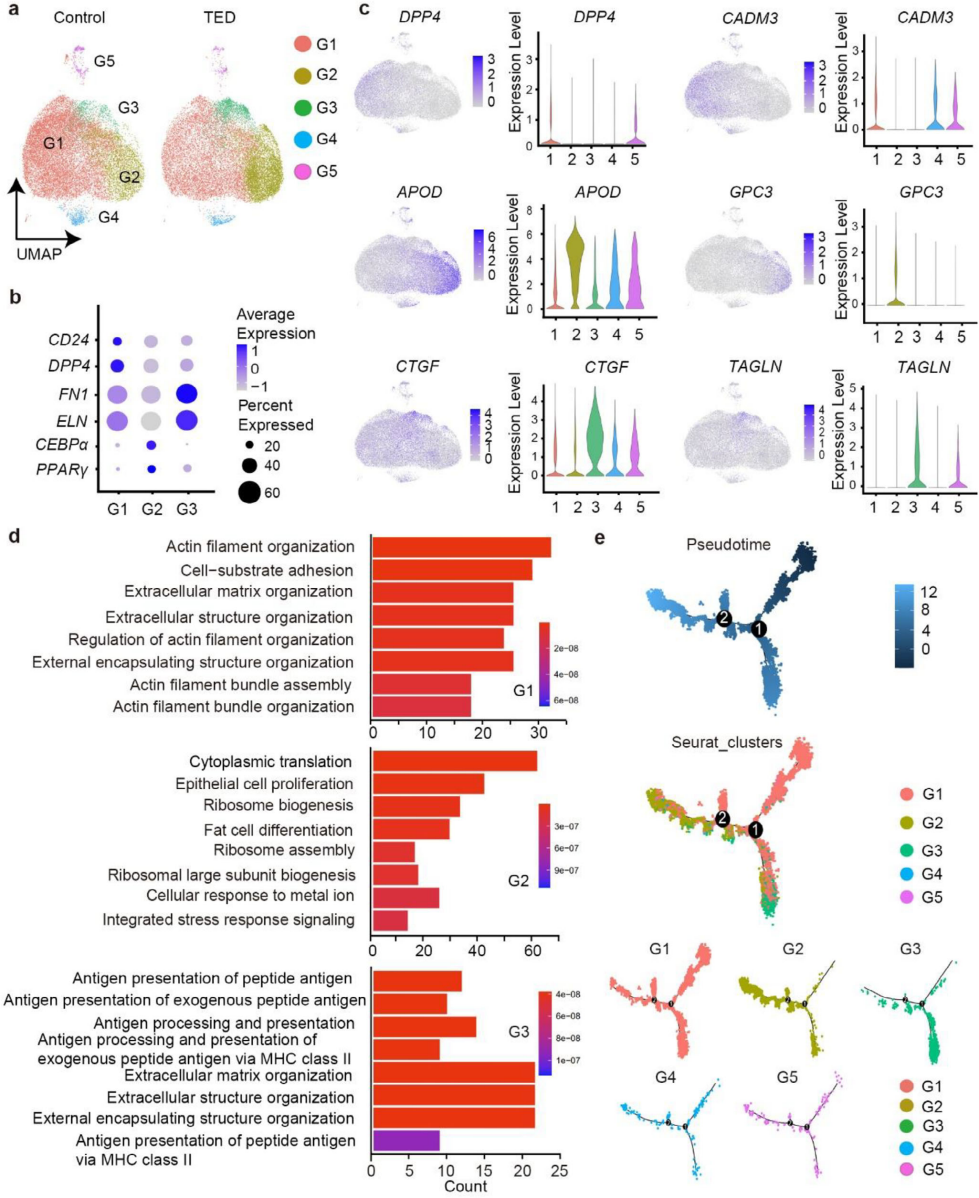
PDGFRα^+^DPP4^+^ fibroblasts are progenitors with enhanced fibrogenic capacity in TED. a) UMAP plot shows five diverse fibroblast subgroups labeled as G1–G5 after unsupervised clustering of fibroblasts from both control‐derived and TED‐derived orbital adipose SVF. All scRNA‐seq analyses presented in the figure are based on a total of five SVF samples, which include two control and three TED‐associated SVF samples. b) Dot plot showing the expression of representative markers of progenitor cells (*CD24* and *DPP4*), fibrosis (*ELN* and *FN1*), and adipogenesis (*PPARG* and *FABP4*) in the G1 to G3 subgroups. Dot size represents the proportion of cells expressing the denoted gene. Color indicates the average normalized expression level within the cluster. c) The left panel of the feature plot and violin plot shows the distribution and expression levels of representative markers of progenitor cells (*DPP4*), adipogenesis (*APOD*), and fibrosis (*CTGF*) in the G1 to G5 subgroups. The right panel shows three highly expressed marker genes detected in G1 (*CADM3*), G2 (*Glypican 3 (GPC3)*), and G3 (*TAGLN*) on a UMAP diagram and a violin plot. The *y*‐axis of the violin plot represents the normalized read count. d) Biological process results of gene ontology enrichment analysis in G1 to G3 sorted by *p*‐adjust < 0.05. e) Pseudotime trajectories of fibroblast subgroups G1 to G5 (upper), colored by Seurat clusters (middle) and split into individual groups (bottom) using Monocle 2. The pseudotime trajectory map is shown from dark to light blue.

To further identify the progenitors of fibrosis in OAT of patients with TED, we re‐clustered 19 882 OAT‐derived fibroblasts from two control subjects and 18 295 OAT‐derived fibroblasts from three TED patients into five groups (G1 to G5) in UMAP (**Figure** **2**a and Figure S2a,b, Supporting Information). Dot plot imaging showed that canonical progenitor markers CD24^[^ ^50^ ^]^ and dipeptidyl peptidase‐4 (DPP4)^[^ ^43^ ^]^ were predominantly expressed in G1, whereas other fibrotic genes, *FN1* and *ELN*, were predominantly expressed in G3. Adipocyte‐associated genes *CEBPα* and *PPARγ* ^[^ ^51^, ^52^, ^53^ ^]^ were predominantly expressed in G2 (Figure 2b). In contrast to DPP4, other common progenitor and mesenchymal cell surface markers, including CD81 and CD34, showed expression across clusters G1‐ G5 in both control and TED groups (Figure S2c, Supporting Information). The canonical TED marker *THY1* was predominantly expressed in clusters G1, G3, G4, and G5 within the TED group (Figure S2c, Supporting Information). Therefore, we defined G1 cells as “progenitors” marked by the expression of *DPP4* and *CADM3* (Figure 2c). G2 was classified as “preadipocytes” with high expression of *Apolipoprotein D (APOD)* and *Glypican 3 (GPC3)*, and G3 was classified as “fibrotic cells” with high expression of representative fibrosis markers *connective tissue growth factor (CTGF)* and *TAGLN* (Figure 2c). Compared with the control group, TED patient‐derived fibroblasts exhibited decreased G1 progenitors (76.1%–54.2%) while increased G2 preadipocytes (15.9%–33.7%) and G3 fibrotic cells (4.3%–9.4%).

Gene‐ontology enrichment analysis of cluster‐specific differentially expressed genes (DEGs) linked G1 and G3 to ECM organization, while G2 was associated with adipocyte differentiation (Figure 2d), highlighting fibroblast diversity. Pseudotime trajectories analysis revealed that fibroblasts primarily arose from G1, with subsequent differentiation predominantly towards G2 or G3 states, implicating G1 as a progenitor for both preadipocytes and fibrotic cells. Consistent with this, G1 abundance was reduced, whereas G2 and G3 abundance were increased in TED patient OATs versus non‐TED controls, indicating enhanced differentiation of PDGFRα^+^DPP4^+^ progenitors into these fates in the TED context. Additionally, cells from clusters G4 and G5 were observed along multiple pseudotime trajectories (Figure 2e).

### 2.3 PDGFRα^+^DPP4^+^ Fibroblasts in OAT Display Significant Fibrogenic Capacity in TED

**Figure 3:**
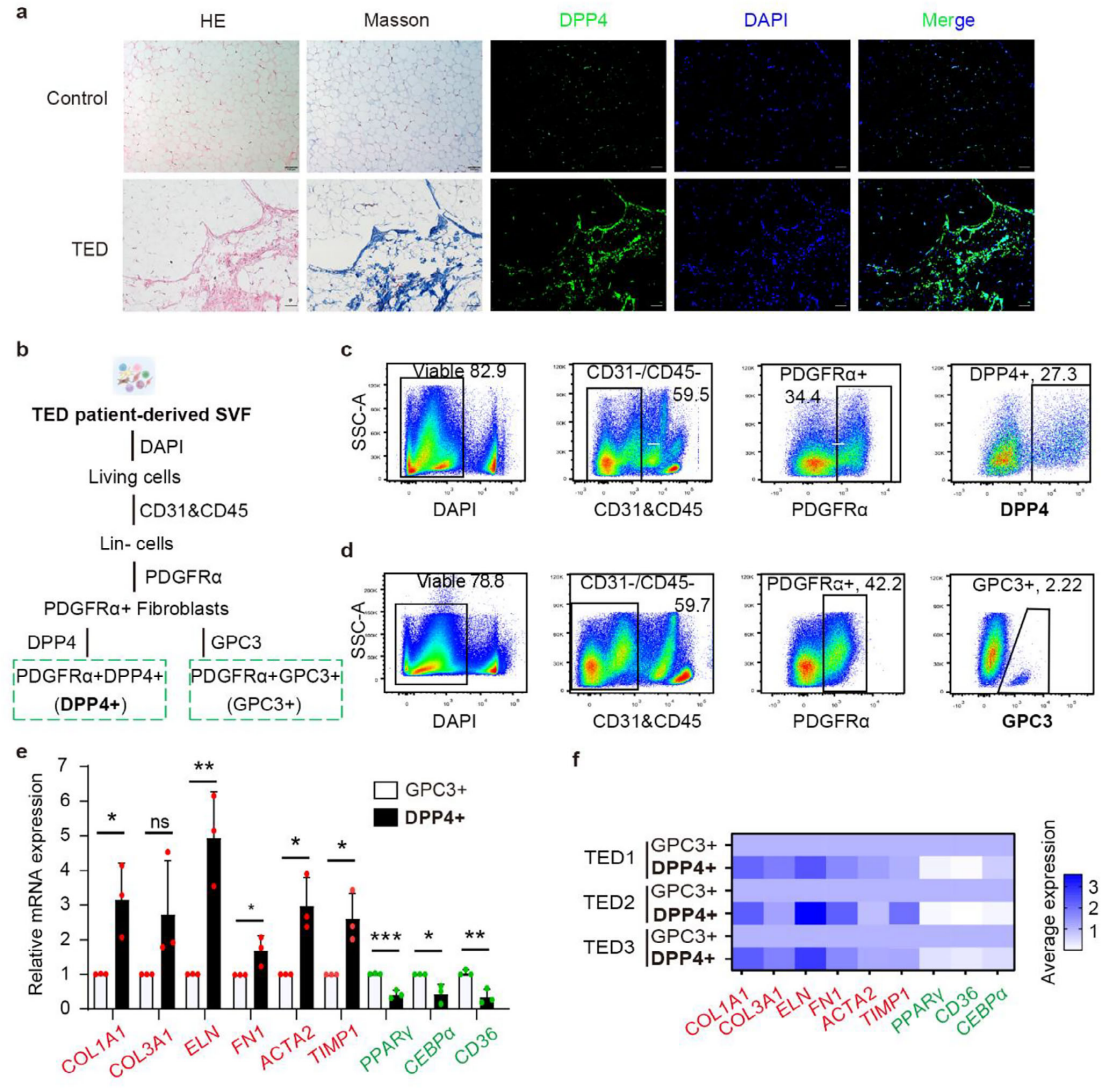
PDGFRα^+^DPP4^+^ fibroblasts within OAT display significant fibrogenic capacity in TED. a) Hematoxylin and eosin (H&E), Masson's trichrome (Masson), and immunofluorescence staining of DPP4 (green) and DAPI (blue) in control and TED serial section tissue samples (scale bar = 100 µm, *n* = 3 patients). b) Schematic of experimental design for flow cytometry. DPP4^+^ cells indicate PDGFRα^+^DPP4^+^ fibroblasts, and GPC3^+^ cells indicate PDGFRα^+^GPC3^+^ fibroblasts in all the figures. c,d) Isolation results of c) PDGFRα^+^DPP4^+^ fibroblasts and d) PDGFRα^+^GPC3^+^ fibroblasts from SVF by flow cytometry. e) qRT‐PCR assay results of fibrosis‐ (red) and adipogenesis‐ (green) associated marker gene expression in PDGFRα^+^DPP4^+^ and PDGFRα^+^GPC3^+^ fibroblasts isolated via flow cytometry. Data are presented as mean ± standard deviation (SD) (*n* = 3, representing three biologically independent experiments conducted using three different patient‐derived cells). Two‐sided unpaired *t*‐test, *p < 0.05, **p < 0.01, ***p < 0.001. f) Single‐cell RNA sequencing showing similar expression patterns of fibrosis‐ (red) and adipogenesis‐ (green) marker genes in the SVF of OAT from three patients with TED.

To investigate the pro‐fibrotic function of G1 cells, we first assessed spatial distribution using tissue staining of OAT from patients with TED and control subjects. Hematoxylin and eosin (H&E) and Masson's trichome staining revealed significant fibrosis, as well as collagen deposition, in the TED group compared with the control group (**Figure** **3**a). Serial sections of OAT with DPP4 fluorescent staining revealed the topographical distribution of G1 progenitors in the fibrotic area in the TED group, whereas the control group exhibited a more diffuse distribution of G1 progenitors. We subsequently examined the fibrogenic potential of G1 cells by isolating living PDGFRα^+^DPP4^+^ and PDGFRα^+^GPC3^+^ cells from the TED patient‐derived SVF cells using FACS (Figure 3b–d) and measuring the expression of signature genes relevant to fibrosis and adipogenesis (Figure 3e). Consistent with the scRNA‐seq expression results, PDGFRα^+^DPP4^+^ cells exhibited high expression of fibrosis markers at the transcriptional level, whereas PDGFRα^+^GPC3^+^ cells expressed high levels of adipogenesis‐associated markers (Figure 3e,f). These functional data confirm that PDGFRα^+^DPP4^+^ cells, also defined as G1 cells, have significant fibrogenic capacity in TED.

### 2.4 Macrophages Activate PDGFRα^+^DPP4^+^ Fibroblasts to Promote Fibrosis

**Figure 4:**
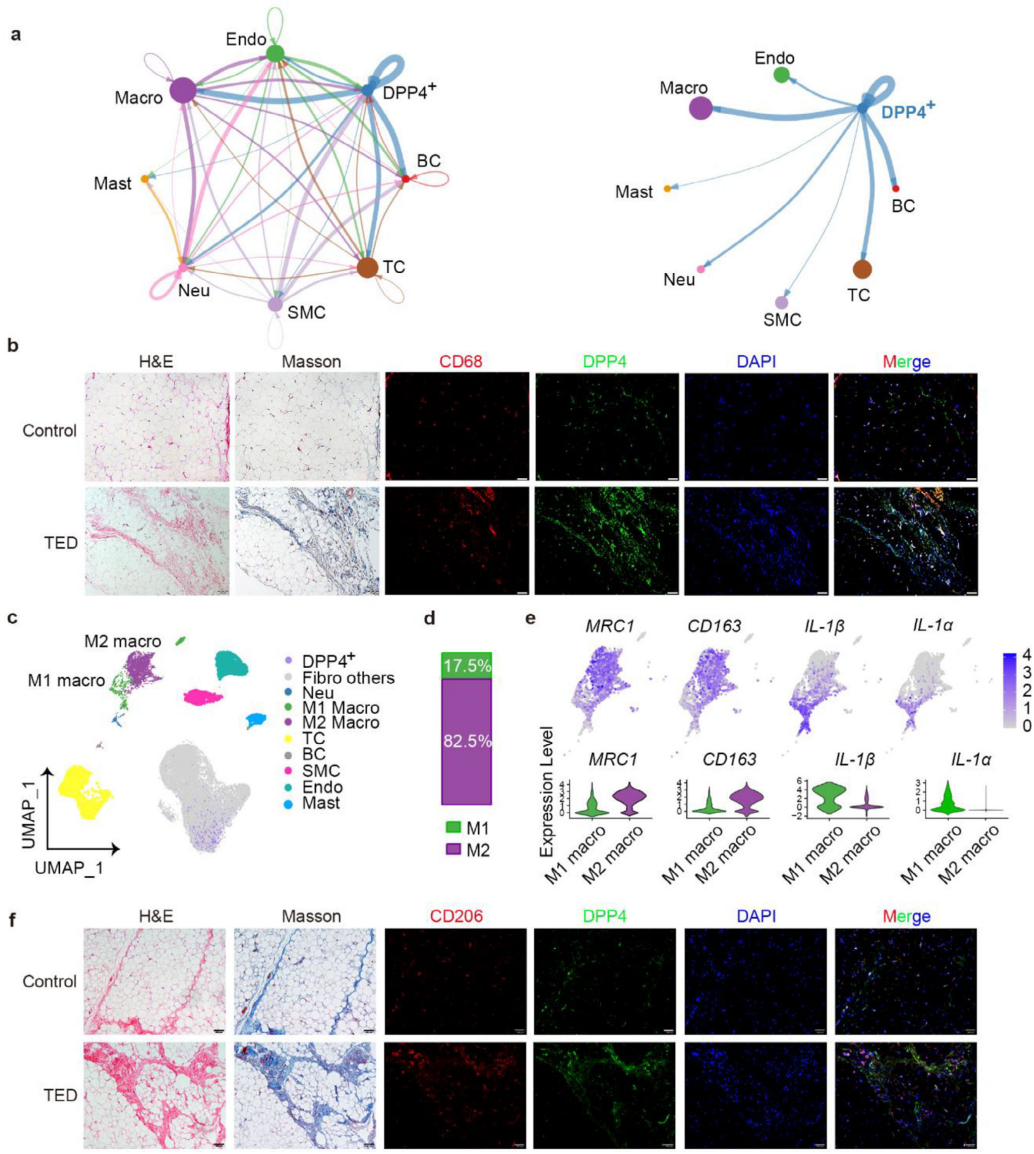
PDGFRα^+^DPP4^+^ fibroblasts accumulate in fibrotic zones of the TED orbital adipose with macrophages. a) CellChat analysis infers cell–cell interactions and cross‐talk strength within the TED‐derived SVF subtypes. The left panel predicts overall interactions; the right panel focuses on PDGFRα^+^DPP4^+^ cells (labeled DPP4^+^ in the figure). Line width indicates the strength of interaction between subclusters. Significant interactions (L–R pairs) with a value > 10 and *p* < 0.05 are shown. b) Representative images showing H&E, Masson, and immunofluorescence staining for CD68 (red), DPP4 (green), and DAPI (blue) in serial sections from OAT from control and TED samples (scale bar = 100 µm, *n* = 3 patients). c–e) Characteristics of macrophages from OAT in patients with TED. (c) UMAP of SVF cells from the OAT of three patients with TED, colored by subgroup identity. Each dot represents a single cell. d) Proportions of M1/M2 macrophage subgroups from TED samples. e) Feature plot and violin plot showing the representative marker expression in M1/M2 macrophage subgroups. f) Representative images of H&E, Masson, and immunofluorescence staining of CD206 (red), DPP4 (green), and DAPI (blue) in serial sections of OAT from control and TED samples (scale bar = 100 µm, *n* = 3 patients).

To further assess the potential trigger of fibrosis in PDGFRα^+^DPP4^+^ progenitors, we predicted cell–cell interaction network analyses using CellChat following re‐clustering SVF cells isolated from the TED group (Figure S3a,b, Supporting Information). The analysis highlighted extensive, putative communication networks encompassing all SVF cell types within the TED context (**Figure** **4**a). Interestingly, CellChat inferred that macrophages exhibit the strongest interaction with PDGFRα^+^DPP4^+^ cells (Figure 4a). We therefore investigated the colocalization of macrophages and PDGFRα^+^DPP4^+^ cells using immunofluorescence of serial sections of OAT in patients with TED and control subjects and found that Cluster of Differentiation 68 (CD68)‐labeled macrophages and DPP4‐labeled PDGFRα^+^DPP4^+^ cells have similar topographical distribution in the fibrotic area (Figure 4b).

The macrophages in the OAT of patients with TED can be further classified into M1‐like (highly expressing *IL‐1α* and *IL‐1β*) and M2‐like (highly expressing *Mannose receptor C‐Type 1 (MRC1)* and *CD163*) populations, and the latter represents the vast majority (82.5%) (Figure 4c–e). By analyzing serial sections of orbital adipose tissue (OAT) via immunofluorescence in both TED patients and controls, we observed that CD206‐labeled macrophages exhibited a comparable topographical distribution with PDGFRα^+^DPP4^+^ cells, showing a similar spatial arrangement predominantly within fibrotic areas (Figure 4f). This localization was consistent with that of CD68‐labeled macrophages.

**Figure 5:**
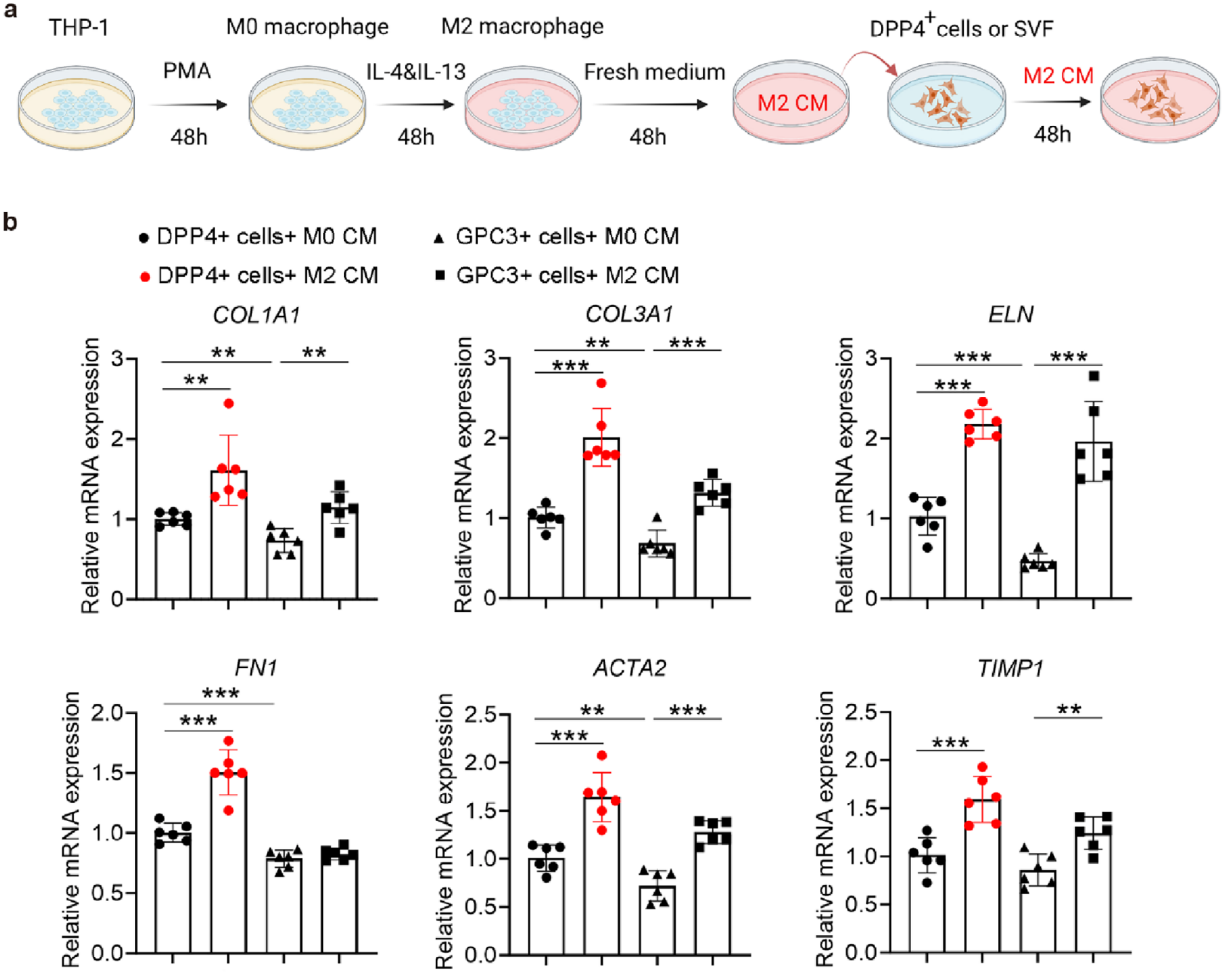
M2 macrophage‐induced PDGFRα^+^DPP4^+^ cell fibrosis in vitro. a) Schematic representation of an in vitro experimental investigating the interaction between PDGFRα^+^DPP4^+^ cells (isolated from patients' SVF, labeled DPP4^+^ in the figure) and THP‐1 induced M2 macrophage. Utilizing PDGFRα^+^GPC3^+^ cells (isolated from patients SVF, labeled GPC3^+^ in figure) as control. b) qRT‐PCR analysis results of fibrosis‐associated gene expression of PDGFRα^+^DPP4^+^ fibroblasts and PDGFRα^+^GPC3^+^ cells after treatment with the supernatant of M2 or M0 macrophages for 48 h. M0 macrophage‐conditioned medium was used as control. Data are presented as mean values ± standard deviation (SD) (*n* = 6, representing six biologically independent experiments conducted using six different patient‐derived cells). Two‐sided unpaired *t*‐test, **p < 0.01, ***p < 0.001.

To determine whether M2 macrophages could induce fibrosis of PDGFRα^+^DPP4^+^ from TED patients, we treated them with the conditioned medium of M2 macrophages originating from the THP‐1 cells (**Figure** **5**a). We found that the conditioned medium treatment induced the expression of fibrosis‐associated markers, including *COL1A1, COL3A1, ELN*, and *FN1*,^[^ ^46^, ^47^, ^54^ ^]^ myofibroblast marker *ACTA2*,^[^ ^55^, ^56^ ^]^ and ECM‐degradation inhibitory factor *TIMP1* ^[^ ^57^ ^]^ in PDGFRα^+^DPP4^+^ cells (Figure 5b). In comparison, the PDGFRα^+^GPC3^+^ cells from TED patients, as well as the PDGFRα^+^DPP4^+^ cells and PDGFRα^+^GPC3^+^ cells from control subjects, exhibited an increase in fibrosis‐associated markers upon exposure to the conditioned medium of M2 macrophages, albeit to a lesser extent (Figure 5b and Figure S4a, Supporting Information). Similarly, but to a lesser extent, phenotypes were observed in the SVF cells of TED after M2‐conditioned medium treatment (Figure S4b, Supporting Information). These results suggest that M2 macrophages may secrete certain factors that precipitate fibroblast fibrosis, a phenomenon that is likely dependent on the spatial distribution of the interacting cell types.

### 2.5 Macrophages Interact with PDGFRα^+^DPP4^+^ Fibroblasts via the GAS6‐AXL Pathway

**Figure 6:**
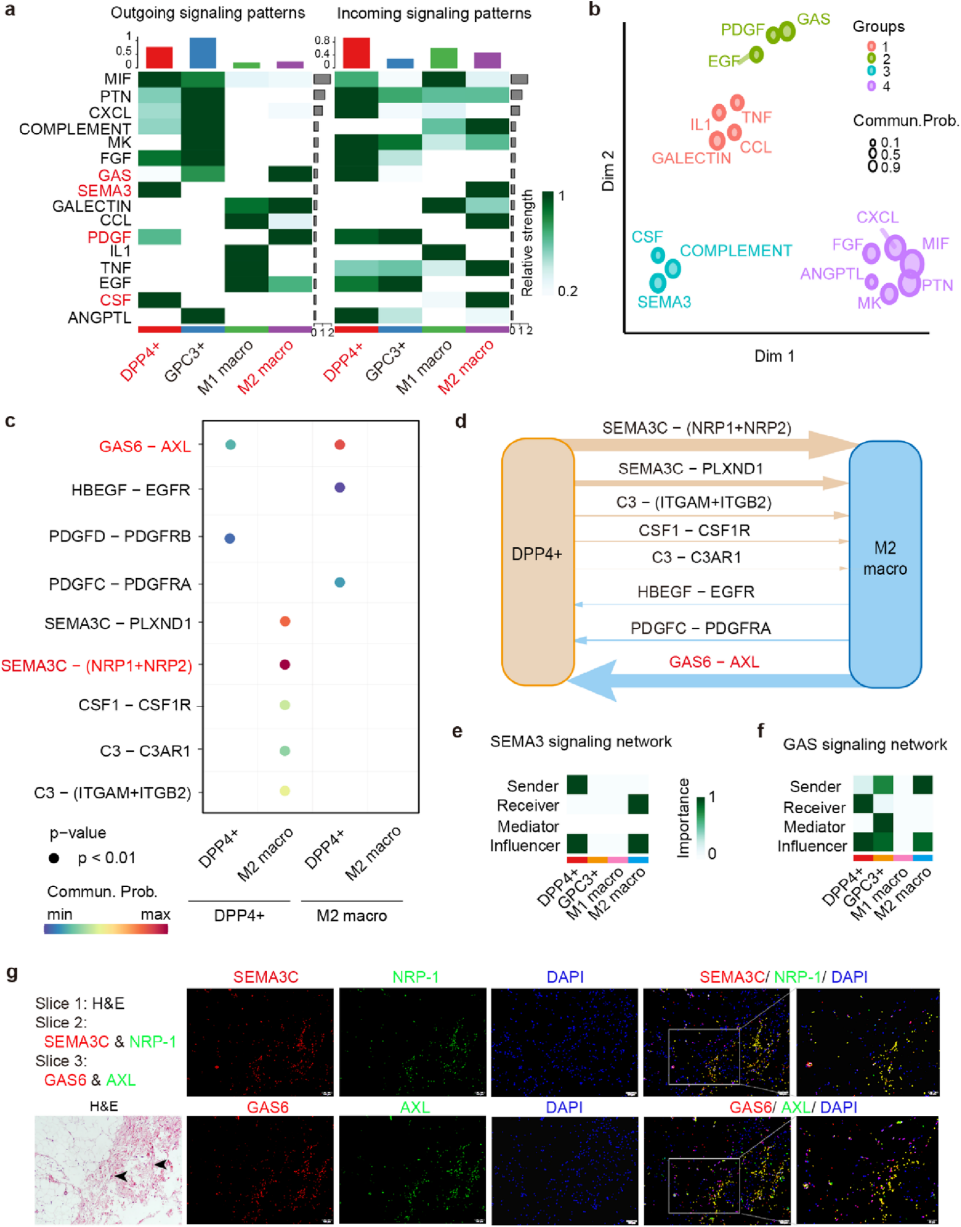
Signaling interactions reveal that macrophages may activate PDGFRα^+^DPP4^+^ fibroblasts via the GAS6‐AXL pathway. a) Cell‐Chat analysis results of potential interactions between PDGFRα^+^DPP4^+^ or PDGFRα^+^GPC3^+^ fibroblasts and M1/M2 macrophage subgroups. Heatmap shows the inferred outgoing communication patterns of secreting cells (left) and the incoming communication patterns of receiving cells (right), revealing corresponding inferred latent patterns and cell groups, as well as signaling pathways. b) Jointly projecting and clustering signaling pathways from fibroblasts and macrophages into a shared two‐dimensional manifold according to their functional similarity. Circles represent the signaling networks from fibroblast and macrophage subgroups, and each circle represents the communication network of one signaling pathway. Circle size is proportional to the total communication probability (Commun. Prob). Different colors represent different groups of signaling pathways. c) Bubble plot shows selected L–R pairs of PDGFRα^+^DPP4^+^ cells and M2 macrophages. The colors range from blue to red, representing low to high communication probability. The bubble size represents the corresponding *p*‐values. d) Overall pathways involved in cross‐talk between PDGFRα^+^DPP4^+^ fibroblasts and M2 macrophages. The line width indicates the interaction strength. e,f) Heatmap showing the importance of each cluster, based on four network centrality measures, of e) SEMA3 and (f) GAS signaling. g) Representative serial‐section images of H&E staining (Slice 1), immunofluorescence staining of SEMA3C/NRP‐1 (Slice 2), and GAS6/AXL (Slice 3) in OAT from patients with TED (scale bar = 100 µm, *n* = 3 patients). SEMA3C and GAS6 are stained red; NRP‐1 and AXL are green, and DAPI is blue.

To explore signaling crosstalk between M2 macrophages and PDGFRα^+^DPP4^+^ progenitors, we predicted key outgoing and incoming signals for specific cell types, including PDGFRα^+^DPP4^+^ progenitors, PDGFRα^+^GPC3^+^ preadipocytes, M1, and M2 macrophages using CellChat (**Figure** **6**a). Outgoing signaling for PDGFRα^+^DPP4^+^ cells predominantly included Semaphorin 3 (SEMA3), Colony Stimulating factor (CSF), Macrophage migration inhibitory factor (MIF), and Fibroblast growth factor (FGF), whereas incoming signaling mainly included Pleiotrophin (PTN), C‐X‐C motif chemokine ligand (CXCL), GAS, FGF, Midkine (MK), Angiopoietin like (ANGPTL), and PDGF. For M2 macrophages, outgoing signaling included predominantly GAS, PDGF, GALECTIN, and Epidermal growth factor (EGF), whereas incoming signaling included predominantly COMPLEMENT, SEMA3, C‐C Motif chemokine ligand (CCL), Tumor necrosis factor (TNF), and CSF. Quantification of the similarities between these significant signaling pathways based on the similarities in their cellular functional communication networks revealed four groups (Figure 6b). Inflammatory pathways, such as *TNFα*, *IL*, CCL, and GALECTIN, were dominant in Group 1, largely representing paracrine signaling from M1 macrophages. Group 2, enriched in GAS, PDGF, and EGF signaling, represented the dominant paracrine signaling from M2 macrophages. Group 3, enriched in SEMA3, CSF, and COMPLEMENT pathways, represented signaling from PDGFRα^+^DPP4^+^ progenitors. Group 4, enriched in MK, PTN, CXCL, MIF, FGF, and ANGPTL pathways, represented signaling predominantly from PDGFRα^+^GPC3^+^ preadipocytes.

We next used ligand–receptor analysis to further assess potential molecules mediating the intercellular communication between PDGFRα^+^DPP4^+^ progenitors and M2 macrophages. As shown in Figure 6c,d, ligand–receptor pairs ranked by interaction strength implied that PDGFRα^+^DPP4^+^ progenitors may interact with M2 macrophages through pathways such as SEMA3C‐NRP1/NRP2/PLXND1 for recruitment. Macrophages might subsequently affect PDGFRα^+^DPP4^+^ progenitors via pathways like the GAS6‐AXL and PDGFC‐PDGFRA pathways (Figure 6c,d). CellChat assessment further indicated that, in the SEMA3 signaling network, PDGFRα^+^DPP4^+^ progenitors act as senders to the receiver M2 macrophages (Figure 6e). In the GAS signaling interactions, M2 macrophages act as senders, mainly secreting GAS to crosstalk with PDGFRα^+^DPP4^+^ cells (Figure 6f). To validate these possible interactions, we examined the physical distribution of these ligand–receptor pairs using continuous section H&E staining and immunofluorescence staining of SEMA3C‐NRP1 and GAS6‐AXL, respectively (Figure 6g). The results indicated that these ligand–receptor pairs have close spatial distribution around the fibrotic area of OAT in TED.

### 2.6 Inhibition of Macrophage‐Mediated Fibrosis by AXL‐Specific Inhibitor TP0903 or AXL Knockdown

**Figure 7:**
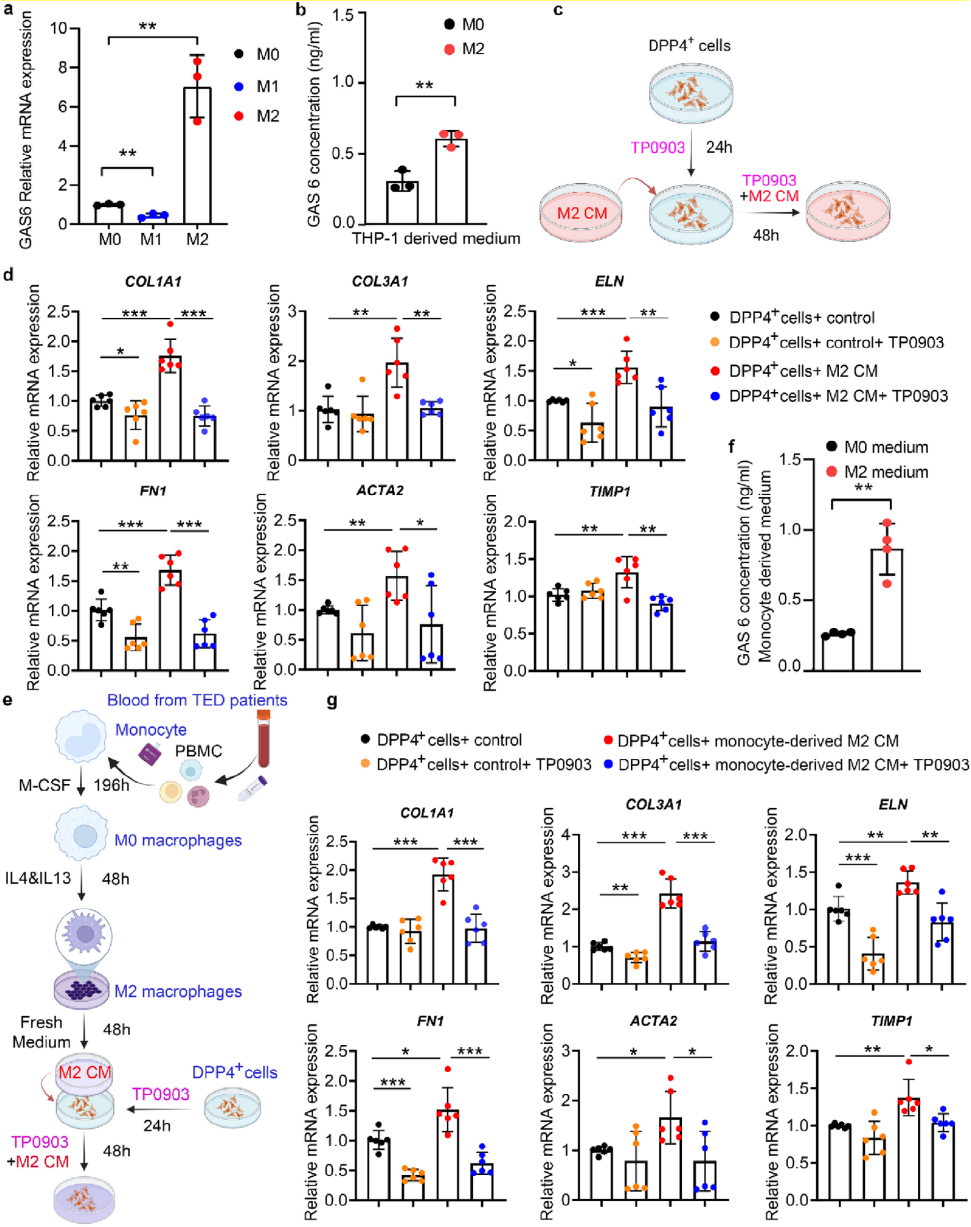
AXL inhibitor TP0903 inhibits fibrosis in PDGFRα^+^DPP4^+^ fibroblasts induced by M2 macrophages derived from THP‐1 cells and from TED patients. a) Comparison of relative GAS6 mRNA expression in M0 macrophages, LPS‐induced M1 macrophages, and IL‐4 and IL‐13‐induced M2 macrophages derived from THP‐1 cells. Data are presented as mean ± standard deviation (SD) (*n* = 3). Two‐sided unpaired *t*‐test, **p < 0.01. b) ELISA results of GAS6 concentration in the supernatant of M0 and M2 macrophages derived from THP‐1 cells. Data are presented as mean ± SD (*n* = 3). Two‐sided unpaired *t*‐test, **p < 0.01. c) Schematic representation of in vitro experiment verifying our hypothesis that blocking GAS6‐AXL signaling with TP‐0903 pre‐treatment reduces M2 macrophage‐mediated fibrosis. d) qRT‐PCR analysis of mRNA expression of fibrosis‐associated genes *COL1A1, COL3A1, ELN, FN1, ACTA2*, and *TIMP1* in PDGFRα^+^DPP4^+^ fibroblasts after treatment with M2 or M0 macrophage conditioned medium, with or without TP0903. M0 macrophage‐conditioned medium was used as a control. Data are presented as mean ± SD (*n* = 6, representing six biologically independent experiments conducted using six different patient‐derived cells). Two‐sided unpaired *t*‐test, *p < 0.05, **p < 0.01, ***p < 0.001. e) Schematic representation of the experimental design to investigate the interaction between PDGFRα^+^DPP4^+^ fibroblasts and monocyte‐derived M2 macrophages in vitro. f) ELISA results of GAS6 concentration in the supernatant of M0 and M2 macrophages, derived from monocytes of patients with TED. Data are presented as mean ± SD (*n* ≥ 3, representing three biologically independent experiments conducted using three different patient‐derived cells). Two‐sided unpaired *t‐*test, **p < 0.01. g) qRT‐PCR analysis of mRNA expression of fibrosis‐associated genes *COL1A1, COL3A1, ELN, FN1, ACTA2*, and *TIMP1* in PDGFRα^+^DPP4^+^ cells after treatment with monocyte‐derived M2 or M0 macrophage conditioned medium with or without TP0903. M0 macrophage‐conditioned medium was used as control. Data are presented as mean ± SD (*n* = 6, representing six biologically independent experiments conducted using six different patient‐derived cells). Two‐sided unpaired *t‐*test, *p < 0.05, **p < 0.01, ***p < 0.001.

To verify whether M2 macrophages stimulate fibrosis of PDGFRα^+^DPP4^+^ cells via the GAS6‐AXL pathway, we determined the expression and secretion of GAS6 in M2 macrophages. GAS6 was specifically induced in the M2 macrophages but repressed in the M1 macrophages (**Figure** **7**a), supporting its increased production by M2 macrophages (Figure 7b). We subsequently tested whether macrophage‐derived GAS6 promotes fibrosis of PDGFRα^+^DPP4^+^ fibroblasts by using a specific AXL inhibitor or anti‐AXL siRNA (Figure 7c,d and Figure S5, Supporting Information). Although AXL has been studied in thyroid‐related cancer, such as papillary thyroid carcinoma, as well as in fibrotic diseases, including pulmonary, hepatic, and intestinal fibrosis, its role in ocular biology remains unexplored.^[^ ^58^, ^59^, ^60^, ^61^ ^]^ Currently, TP0903, a specific inhibitor of AXL,^[^ ^62^, ^63^ ^]^ is in a phase II clinical trial for the treatment of acute myeloid leukemia (NCT03013998), with no direct evidence of application in any fibrotic diseases. In our study, TP0903 significantly reduced the induction of fibrosis markers, such as *COL1A1, COL3A1, ELN, FN1, ACTA2*, and *TIMP*, by THP1‐derived M2 macrophage‐conditioned medium in PDGFRα^+^DPP4^+^ cells compared to controls (Figure 7d and Figure S5a–c, Supporting Information). Besides, the transgenic experiments using AXL knockdown DPP4^+^ fibroblasts (Figure S5d,e, Supporting Information) showed that AXL knockdown in TED‐derived PDGFRα^+^DPP4^+^ cells could also prevailingly inhibit the induction of fibrosis markers by THP1‐derived M2 macrophage‐conditioned medium (Figure S5f, Supporting Information), which is consistent with the pharmacologic results of TP0903. These results indicate that the GAS6‐AXL pathway is required for M2 macrophages to promote fibrosis of PDGFRα^+^DPP4^+^ cells in vitro.

To further determine whether macrophages derived from patients with TED could also stimulate fibrosis of PDGFRα^+^DPP4^+^ cells, we prepared macrophages from isolated peripheral blood mononuclear cells of patients with TED (Figure 7e). We found that the GAS6 concentration in the medium of M2 macrophages derived from patients with TED was 0.8 ng/mL, which was significantly higher than that in the M0 samples (Figure 7f). Consistently, the medium with M2 macrophages derived from patients with TED upregulated fibrosis signature genes in PDGFRα^+^DPP4^+^ cells (Figure 7g). Again, TP0903 treatment abrogated the induction of these fibrotic genes by the medium with M2 macrophages derived from patients with TED. Together, these results indicate that macrophages promote fibrosis in in vitro systems derived from patients with TED via the GAS6‐AXL pathway.

### 2.7 TP0903 Inhibits Fibrosis in Orthotopic Xenograft Models Derived from TED Patients

**Figure 8:**
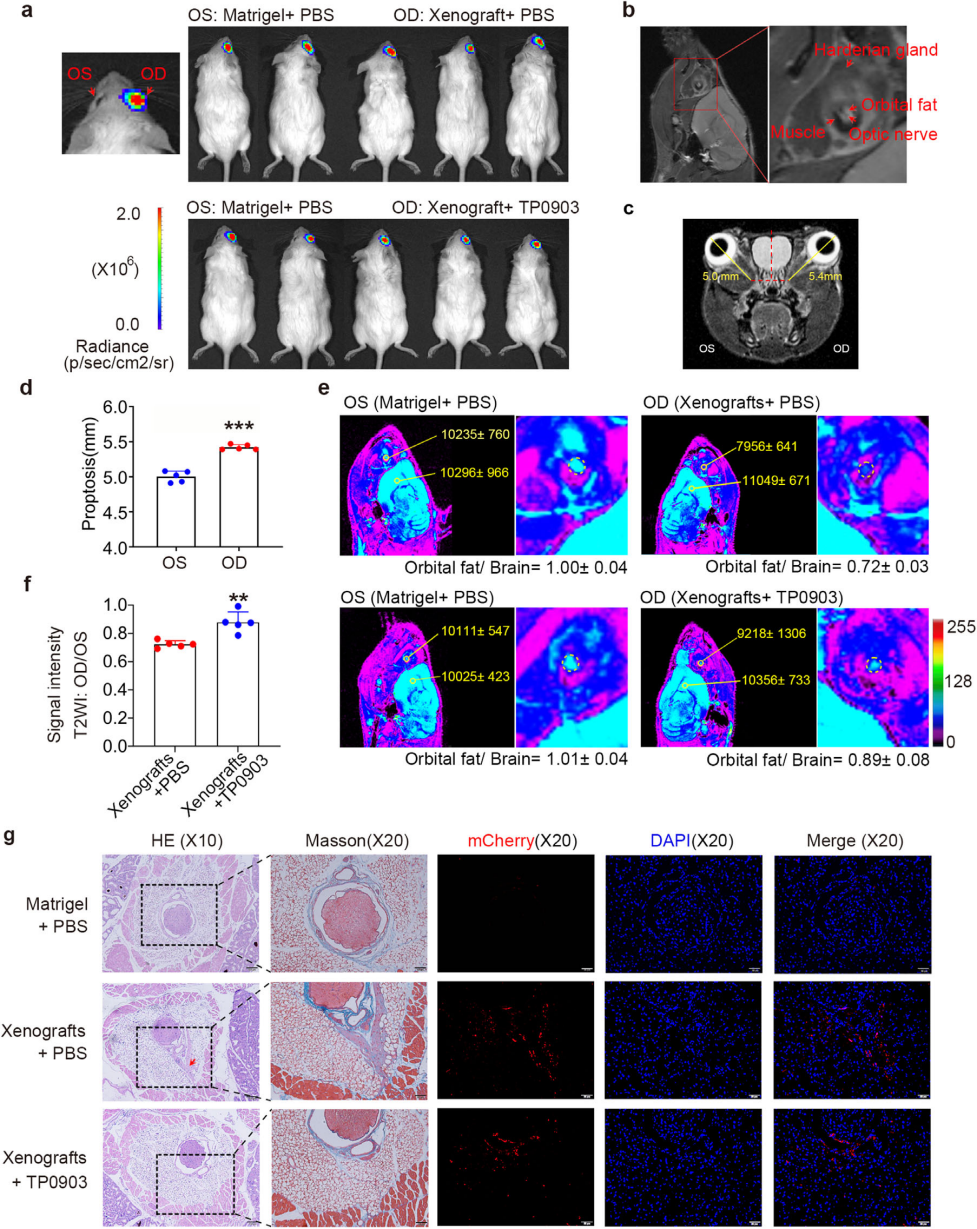
TP0903 inhibits fibrosis in a TED patient cell‐derived orthotopic xenograft model. a) Orthotopic xenografts were established in the right orbits (oculus dexter, OD) of mice by injecting TED patient‐derived orbital fibroblast cell line (OF‐CL) cells expressing mCherry and luciferase, pretreated with either PBS (Xenografts) or TP0903 (Xenografts + TP0903). As controls, the left orbits (oculus sinister, OS) received Matrigel injections (Matrigel + PBS). In vivo luciferase imaging on Day 7 confirmed the orthotopic placement and growth of the xenografts. b) Representative T2‐weighted magnetic resonance imaging (MRI) of the living mouse orbit (left panel) and enlarged view indicating the intra‐orbital structures (right panel). c) Orthotopic xenografts were established in the right mouse orbits (OD) via injection of TED patient‐derived OF‐CL cells, while the left orbits (OS) received Matrigel injections as a control. Axial MRI images revealed increased proptosis in the experimental right eye compared to the control left eye. d) Statistical analysis of proptosis of bilateral eyes following intra‐orbital injections. Data are presented as mean ± standard deviation (SD) (*n* = 5). A two‐sided paired *t‐*test was performed, ***p < 0.001. e) Typical pseudo‐color orbital MRI images of orthotopic xenograft models are shown. Experimental right eyes were injected intra‐orbitally with patient‐derived OF‐CL cells pretreated with PBS (Xenografts + PBS) or TP0903 (Xenografts + TP0903). The signal intensity (mean ± SD) within regions of interest (yellow circles) in orbital fat and the ipsilateral brain is measured. Signal intensity ratios (Orbital Fat/ Brain) were calculated using the ipsilateral brain white matter as reference. Orbital fibrosis was quantified by comparing relative signal intensity between the right (OD) and left (OS) orbits. f) Statistical analysis of the OD/OS signal intensity _(Orbital Fat/ Brain)_ between the xenograft models with or without TP0903 treatment. Data are presented as mean ± SD (*n* = 5). Two‐sided unpaired *t‐*test, **p < 0.01. g) Representative images of continuous section H&E, Masson, and immunofluorescence staining for mCherry (with DAPI) from the injection site within orbital tissues. Rows correspond to the following groups: control group (Matrigel + PBS), xenografts group (Xenografts + PBS), and xenografts pretreated with TP0903 group (Xenografts + TP0903). Each row represents the injection area of intra‐orbital tissues with H&E staining magnified at ×100, Masson staining magnified at ×200, mCherry and DAPI staining magnified at ×200, respectively (*n* = 4). Fibrotic tissue formation within orbital fat is denoted by a red arrow.

To evaluate the anti‐fibrotic effects of TP0903 in vivo, orthotopic mouse models of fibrosis TED were obtained via intra‐orbital injection of GAS6‐pretreated orbital fibroblast cell line (OF‐CL) cells, a fibroblast cell line established from OAT of patients with TED.^[^ ^64^ ^]^ In this experiment, GAS6‐pretreated OF‐CL cells mixed with Matrigel were injected into the right orbital cavity (oculus dexter, OD) of mice to form the xenograft group, whereas an equal volume of PBS mixed with Matrigel was injected into the left orbital cavity (oculus sinister, OS) to serve as the control group (**Figure** **8**a). To track cell survival and location, OF‐CL were co‐expressed with luciferase and mCherry. As shown in Figure 8a, the luciferase‐mCherry dual‐labeled cell line enabled real‐time monitoring of cell distribution within the orthotopic mouse models. Luciferase in vivo imaging on Day 7 showed that the xenografts were orthotopically placed in the orbit and had survived to grow. Consistent with previous studies,^[^ ^65^, ^66^ ^]^ magnetic resonance imaging (MRI) could visualize the structure of the OAT, as well as the optic nerve, extraocular muscle, and the harderian gland in the orbit of the mice (Figure 8b). Axial MRI images showed increased proptosis in the PBS‐pretreated xenograft group (Figure 8c,d), consistent with the exophthalmos observed in patients with TED. To assess the in vivo anti‐fibrotic effects of TP0903, OF‐CL cells pretreated with GAS6 were further treated with or without TP0903 before being injected into the right cavities of the mouse (Figure 8a). Typical pseudo‐color orbital MRI images of the bilateral sides were shown (Figure 8e). The OAT without fibrosis exhibited high signal intensity, whereas the fibrotic tissues were characterized by low signal intensity on T2‐weighted MRI. A region of interest (ROI) circle was placed within the orbital fat region to measure the signal intensity, and this measured signal intensity was set in proportion to that of the ipsilateral brain as reference (Orbital Fat/Brain). The signal intensity ratio for each mouse was further calculated by taking the ratio of the xenograft eye (OD Orbital Fat/Brain) to the control eye (OS Orbital Fat/Brain) to account for individual differences. The OD/OS signal intensity_(Orbital Fat/Brain)_ ratios in the mice ranged from 0.69 to 0.76 for the xenograft group pre‐treated with PBS, and from 0.79 to 0.99 for the xenograft group pre‐treated with TP0903 (Figure 8e,f). Comparison between the two groups showed a significant increase in OD/OS signal intensity_(Orbital Fat/Brain)_ ratio in the TP0903‐pretreated xenograft group, indicating that TP0903 suppresses orbital fibrosis (Figure 8f). H&E, Masson, and Collagen I immunofluorescence staining of serial sections confirmed the formation of fibrotic tissue within the OAT in the PBS‐pretreated xenograft group, whereas TP0903 pre‐treatment inhibited fibrosis (Figure 8g and Figure S6, Supporting Information). No obvious histopathologic changes were observed in the control Matrigel group (Figure 8g). Immunofluorescence staining of mCherry showed that the OF‐CL cells were transplanted and maintained viability within the OAT area of mice, with their distribution coinciding with the fibrotic regions. Conversely, in the TP0903‐group, although mCherry‐labeled cells were detectable, the fibrotic areas (as indicated by Masson's trichrome staining) and Collagen I expression were notably reduced compared to the PBS‐pretreated xenograft group (Figure 8g and Figure S6, Supporting Information). Moreover, orthotopic xenograft models with TED patient‐derived SVF cells were established to further determine the in vivo function of TP0903 (Figure S7, Supporting Information). Consistently, TP0903 treatment inhibited OAT fibrosis induced by intra‐orbital injection of patient‐derived SVF cells (Figure S7, Supporting Information). The above results collectively demonstrate that TP0903 displays an anti‐fibrotic function in the TED patient‐derived orthotopic xenograft mouse models.

## 3 Conclusion

The lack of medical treatments for fibrosis as alternatives to surgical intervention remains a significant clinical hurdle in inactive‐stage TED. This study identified PDGFRα^+^DPP4^+^ fibroblasts as progenitor cells implicated in OAT fibrosis within TED. Furthermore, we elucidated a specific immunomodulatory mechanism by which a pathogenic macrophage subset, prevalent in the fibrotic milieu of inactive‐stage TED, promotes fibroblast activation and subsequent fibrosis via the GAS6‐AXL signaling pathway. The functional significance of this pathway was validated in a patient‐derived orthotopic xenograft model of TED, where pharmacological inhibition of AXL with TP0903 yielded substantial anti‐fibrotic effects.

The progenitor cells responsible for fibrosis in TED remain poorly defined, posing challenges for the development of effective anti‐fibrotic therapies. Given the distinct molecular properties of adipose tissue across different body regions^[^ ^67^ ^]^ and their varied responses to pharmaceutical interventions,^[^ ^64^ ^]^ therapeutic strategies must be tailored to account for these differences. In this study, we performed an unbiased scRNA‐seq analysis of the non‐adipocyte fraction of human OAT from patients with or without TED at the inactive, fibrosis‐characterized stage. Among SVF cells, orbital fibroblasts constitute the largest population and serve as key effector cells in OAT fibrosis (Figure 1). These orbital fibroblasts express PDGFRα, indicating a mural cell origin,^[^ ^68^ ^]^ consistent with our previous finding that Lenvatinib, an FDA‐approved Vascular endothelial growth factor receptor (VEGFR) inhibitor, holds potential as a therapeutic agent for TED.^[^ ^69^ ^]^ However, the molecular characteristics of orbital fibroblasts differ from those of fibroblasts in other peripheral adipose tissues, exhibiting lower expression of markers like *PDGFRβ*,^[^ ^32^ ^]^ *CD248*,^[^ ^48^ ^]^ and *ITGBL1*,^[^ ^49^ ^]^ while maintaining high *PDGFRα* expression^[^ ^35^ ^]^ (Figure S1d, Supporting Information). This distinction may reflect the unique developmental, homeostatic, and reparative functions of OAT‐derived fibroblasts in the orbital region. Notably, orbital fibroblasts originate from the neural crest, whereas fibroblasts in other adipose tissue primarily arise from the mesenchymal lineages.^[^ ^70^ ^]^

Our study identified a PDGFRα^+^DPP4^+^ orbital fibroblast subset within the fibrotic regions of OAT in TED patients, exhibiting both fibrotic potential and progenitor‐like transcriptional traits (Figures 2 and 3). We found that CD81^[^ ^42^ ^]^ and CD34,^[^ ^44^ ^]^ markers, commonly associated with adipose progenitors in peripheral adipose tissues, are non‐specifically expressed across orbital fibroblast subgroups in TED patients (Figure S2, Supporting Information). Interestingly, although CD34^+^ cells are broadly expressed across various subpopulations, the expression levels vary significantly among these subsets. In control samples, CD34 is predominantly expressed in progenitor cells. In contrast, in TED samples, its expression begins to increase in preadipocytes and fibrosis‐characterized subpopulations (data not shown). This finding may be associated with previous reports that the CD34^+^ OFs are increased in TED orbits and thought to undergo transition into myofibroblasts or adipocytes, and MiR‐182‐5p promotes the proliferation, migration, fibrosis, and anti‐apoptosis of CD34^+^ OFs via targeting Smad7.^[^ ^12^, ^71^ ^]^ THY1, a surface marker elevated in TED orbital fibroblasts,^[^ ^72^, ^73^ ^]^ delineates functionally distinct subpopulations: THY1^−^ cells are capable of adipogenesis,^[^ ^31^, ^32^ ^]^ while THY1^+^ cells contribute to fibrosis.^[^ ^74^, ^75^ ^]^ Our findings show that THY1 expression is absent in the G2 pre‐adipocyte subpopulation but is prevalent in G1, G3, and G5 orbital fibroblasts (Figure S2, Supporting Information). Conversely, DPP4, a progenitor marker in subcutaneous fat depots,^[^ ^43^ ^]^ is exclusively expressed in the G1 progenitor‐like group (Figure 2 and Figure S2, Supporting Information). However, in our study, the number of DPP4^+^ cells was reduced, whereas fibrotic cells were increased in TED patients compared to non‐TED controls (Figure 2a). This observation supports the notion that DPP4^+^ cells serve as progenitors of fibrotic cells, with an increased propensity to differentiate into myofibroblasts within the TED orbital microenvironment. Indeed, pseudotemporal trajectory analysis using the scRNA‐seq data suggests that G1 cells may transdifferentiate into G3 cells (Figure 2e).

In our study, scRNA‐seq revealed that macrophages show the most significant increase in SVF cells within the TED group compared to controls (Figure 1 and Figure S1, Supporting Information). We also observed substantial intercellular communication between macrophages and PDGFRα^+^DPP4^+^ fibroblasts in TED (Figures 4, 5, 6), reminiscent of the direct macrophage‐fibroblast interactions observed in other peripheral adipose tissues, where macrophages promote fibrosis through the secretion of PDGFs, TGFβ, Amphiregulin (AREG), and IL‐6.^[^ ^76^, ^77^ ^]^ Although Li et al. predicted that RASD1‐expressing lipo‐fibroblasts and macrophages contribute to inflammation and adipogenesis in active‐stage TED using scRNA‐seq,^[^ ^45^ ^]^ Görtz et al. reported hypoxia and M1 macrophage‐orbital fibroblast interactions drive inflammation and adipogenesis in TED.^[^ ^78^ ^]^

Our findings align with previous studies showing that tissue‐resident macrophages with an M2‐like phenotype^[^ ^79^ ^]^ accumulate near target cells to shape a specialized immune microenvironment.^[^ ^29^, ^80^ ^]^ We observed co‐localization of macrophages and PDGFRα^+^DPP4^+^ fibroblasts in the fibrotic regions (Figure 4), suggesting that M2 macrophages participate in orbital fibrosis through direct interactions with PDGFRα^+^DPP4^+^ fibroblasts. It has been noted that DPP4 expression has been reported in macrophages derived from non‐orbital adipose tissue.^[^ ^81^ ^]^ However, our scRNA‐seq data reveal a significant difference in DPP4 expression levels between macrophages and fibroblasts in OAT (Figure S3b,c, Supporting Information). In agreement, immunofluorescence staining for DPP4 and macrophage markers CD68 and CD206 demonstrates distinct DPP4 expression patterns between these two cell types in OAT (Figure 4b,f).

Furthermore, we identified that PDGFRα^+^DPP4^+^ fibroblasts may recruit M2 macrophages by secreting SEMA3C, which interacts with macrophage NRP1/NRP2 receptor. Subsequently, these macrophages promote fibrosis in PDGFRα^+^DPP4^+^ cells via GAS6‐AXL signaling (Figure 6). Notably, the activation of the GAS6/AXL signaling pathway is involved in liver and cardiac fibrosis.^[^ ^82^, ^83^ ^]^ Continuous tissue staining revealed spatial colocalization of ligand–receptor pairs, supporting direct interactions between macrophages and PDGFRα^+^DPP4^+^ fibroblasts (Figure 6g). In vitro, M2 macrophages polarized from THP‐1 cells and TED‐derived monocytes exhibited increased GAS6 secretion (Figure 7). Furthermore, M2 macrophages induced fibrosis in TED‐derived PDGFRα^+^DPP4^+^ cells, a process attenuated by either the AXL inhibitor TP0903 or AXL knockdown (Figure 7 and Figure S5, Supporting Information).

In vivo, orbital fibrosis was significantly mitigated by TP0903 treatment in a patient‐derived orthotopic xenograft mouse model (Figure 8 and Figures S6 and S7, Supporting Information). Given the structural difference in orbital tissues between mice and humans and the lack of reproducible in vivo models for drug discovery for TED,^[^ ^84^, ^85^, ^86^ ^]^ we previously established a stable and easy‐to‐use preclinical platform based on the TED patient‐derived OF‐CL for anti‐adipogenesis drug screening.^[^ ^64^ ^]^ In this study, we further refined the orthotopic xenograft mouse models to facilitate the repurposing of anti‐fibrosis drugs. MRI and luciferase tracing revealed proptosis and increased orbital fibrosis signals, with H&E, Masson, and immunostaining confirming fibrotic tissue in the OAT of xenograft versus control eyes (Figure 8 and Figures S6 and S7, Supporting Information). TP0903 showed in vivo anti‐fibrotic efficacy (Figure 8 and Figures S6 and S7, Supporting Information), highlighting its potential for drug repurposing and the accelerated development of anti‐fibrosis therapies for TED patients.

Certain limitations should be acknowledged. Although the observed effects of GAS6‐AXL targeting in vitro and in our patient cell‐derived orthotopic xenograft model of TED‐associated orbital fibrosis are promising, our model's inability to fully replicate the systemic immune complexity inherent in TED patients is a constraint, given the absence of a validated transgenic animal model for the disease. Consequently, further refinement of our xenograft model to incorporate a more physiologically relevant immune system represents a key future direction for the field, leveraging the insights gained from this study.

In summary, we identified M2 macrophages as the key driver of fibrosis in the orbital microenvironment through their interaction with PDGFRα^+^DPP4^+^ cells via the GAS6‐AXL pathway. Our findings uncover a critical macrophage‐fibroblast crosstalk axis that drives fibrosis in TED. We specifically highlight the GAS6‐AXL pathway as a promising, targeted therapeutic node for mitigating established fibrosis, offering a strong rationale for its further investigation in preclinical and clinical settings.

## 4 Experimental Section

### Study Approval

This study was conducted according to the guidelines for human research as stated in the Declaration of Helsinki. The Institutional Review Board of Shanghai General Hospital approved the research protocol (approval no. 2021KY008), and written informed consent for sample collection was obtained from all patients. Patients were all diagnosed by physical and laboratory examinations at Shanghai General Hospital. All animal experiments were approved by and performed in accordance with the guidelines of the Institutional Review Board and IACUC of Shanghai General Hospital (approval no. 2020AWS0047).

### Clinical Samples

Orbital adipose tissue samples were collected from 28 patients with inactive‐stage TED (clinical activity score < 3) undergoing orbital decompression surgery. Among these patients, peripheral blood samples were additionally obtained from 6 individuals. For comparison, OAT samples from 12 control subjects with orbital fat prolapse were also included in the study. (Table S1). Patients with TED and control subjects were recruited from the Shanghai General Hospital. Patients were all diagnosed by physical and laboratory examinations at Shanghai General Hospital.

### Stromal Vascular Fraction Separation of Orbital Adipose Tissue

Single‐cell suspensions were prepared with OAT from three patients with TED (TED1–3, information provided in Table S1) and two control subjects with prolapsed OAT (CON1–2, information provided in Table S1) by shredding into several fractions, followed by digestion with 175 U mL^−1^ collagenase I (Diamond, China) for at least 1 h at 37 °C. Digestion was terminated by tissue fraction decomposition and the appearance of filamentous fibers. The digested tissue was passed through a 100 µm cell strainer, followed by red blood cell lysis using red blood cell lysate (Beyotime, China), and then passed through a 40 µm cell strainer to remove mature adipocytes and impurities. After centrifugation, the SVF pellet was resuspended in complete medium (10% fetal bovine serum, FBS, with Dulbecco's Modified Eagle Media, DMEM/F12 medium).

### ScRNA‐seq and Quality Control

After SVF extraction, we captured and barcoded approximately 1.5 × 10^4^ living single cells from each patient in 10× Chromium Controller (10× Genomics) as previously reported.^[^ ^87^ ^]^ We subsequently pooled all complementary DNA (cDNA) and constructed a general library, which was sequenced by Illumina. Cell Ranger (10x Genomics, v3.0.1) was used for bcl2fastq conversion, aligning (using the hg38 reference genome), filtering, counting, cell calling, and aggregating. Seurat (v 4.0.4) was used for filtering, UMAP generation, and initial clustering. We included genes expressed in at least three cells, as well as cells with at least 200 genes. nFeature, nCount, and percent.mt were generated to ascertain the filtering criteria of 200 < nFeature < 9500, percent.mt < 15, and nCount < 110 000. After filtering, a total number of 58 253 cells (28 541 from control samples and 29 712 from TED samples) were used for cell clustering. For re‐clustering of the three TED samples, we included genes expressed in at least three cells, as well as cells with at least 200 genes. nFeature, nCount, and percent.mt were generated to ascertain the filtering criteria of 200 < nFeature < 8500, percent.mt < 15, and nCount < 110 000. After filtering, a total number of 29 709 cells were used for cell clustering.

### Cell Clustering and Marker Gene Identification

After completion of quality control, we log‐normalized the data of five samples and identified the top 2000 variable genes using the “vst” selection method. We then linearly transformed (“scaled”) these genes so that for each gene, the mean expression across cells was 0 and the variance across cells was 1. Samples were processed and sequenced in batches. The canonical correlation analysis algorithm was utilized for batch effect correction. We performed dimension reduction, clustering, and differential expression analysis following the Seurat‐guided tutorial. We performed principal component analysis (PCA) of the scaled data using the 2000 variable genes as input. We determined the dimensionality of the data using the elbow plot and used 30 PCs with a resolution of 0.9 to cluster the cells. The main cell clusters were then visualized using UMAP plots. We used the default Wilcoxon rank‐sum test, running the “FindAllMarkers” function in Seurat, to identify differentially expressed markers in each cluster. We then annotated each cell type by searching for the specific gene expression patterns in Human Cell Landscape (http://bis.zju.edu.cn/HCL/landscape.html), CellMarker (http://bio‐bigdata.hrbmu.edu.cn/CellMarker/index.jsp), Human Cell Atlas, and other scRNA‐seq datasets.^[^ ^43^, ^88^ ^]^ Conventional markers described in a previous study were also adopted to categorize every cell into a known biological cell type. For re‐clustering of the three TED samples, we used 30 PCs with a resolution of 3 for cell clustering and detected 52 clusters by UMAP. The following cell annotation was consistent with the standard of five samples.

For re‐clustering of the fibroblasts, we used stricter filtering criteria with 500 < nFeature < 6000, percent.mt < 5, and 1 000 < nCount < 20 000, and a total of 19 882 cells from the control group and 18 295 cells from the TED group, to improve the analysis. We subsequently subset the cells and re‐ran the “FindNeighbors” and “FindClusters” functions, using 30 PCA dimensions for both functions and a resolution of 2.5 for the “FindClusters” function. We visualized the data by recalculating the UMAP plot with the “RunUMAP” function using default parameters and 30 PCA dimensions. To evaluate the differences, we first used the “FindAllMarkers” to identify genes with enriched expression in each fibroblast subcluster separately. We then used “FindMarkers” to compare differentially expressed genes (DEGs) between groups of clusters and merge the clusters without DEGs. For the gene ontology analyses, we queried representative genes expressed by each fibroblast subcluster into GO annotations using the “org.Hs.eg.db” R package (v 3.13) and then used the “enrichGO” function of the clusterProfiler R package (v 4.0.5) for gene ontology analyses. *p*‐values were adjusted by the FDR approach (default *p*‐value < 0.05).

### Pseudotime Analysis

We performed pseudotime analysis of a filtered subset of fibroblast clusters (G1 to G5) using Monocle 2 (v 2.20.0) (http://cole‐trapnell‐lab.github.io/monocle‐release/docs/). By selecting ordering genes using a cut‐off of expression in ≥ 10 cells, and a combination of intercluster differential expression and dispersion with a *q*‐value cut‐off of < 1×10^−10^, we obtained 1087 genes. We visualized the relationship between cell populations and pseudotime by plotting the differentiation trajectory of selected genes exhibiting significant changes through pseudotime or known biologic identity by using the Monocle function “plot cell trajectory”.

### Cell–Cell Communication Analysis

We conducted cell–cell communication analysis using the R CellChat package (v 1.1.3)^[^ ^89^ ^]^ on the TED1–3 samples. Briefly, we identified differentially expressed signaling genes (*p* < 0.05) using the Wilcoxon rank sum test across cell clusters in the scRNA‐seq dataset to calculate the ensemble average expression for communication probability or interaction strength between two cell groups. To identify key signals and latent communication patterns among all the signaling pathways, we applied an unsupervised learning method, non‐negative matrix factorization, to pattern recognition. To evaluate ligand–receptor pairs, we used alluvial plots to show associations between latent patterns and cell clusters and ligand–receptor pairs or signaling pathways. Finally, we used social network analysis to predict signaling sources, influencers, targets, mediators, and higher‐order information.

### Hematoxylin and Eosin, Masson, and Immunofluorescence Staining of OAT

OAT from patients with TED (TED3–7, information provided in Table S1) and control samples (CON3‐9, information provided in Table S1) was fixed with 4% paraformaldehyde overnight, dehydrated with a series of ethanol washes, and subsequently embedded in paraffin for serial sections. These sections were stained with hematoxylin and eosin (H&E) and Masson using standard procedures and examined by light microscopy. We also used the serial sections to analyze the co‐localization of DPP4^+^ cells and macrophages (CD68, CD206), or ligand–receptor pairs SEMA3C‐NRP1 and GAS6‐AXL, with immunofluorescence. Paraffin‐embedded sections were deparaffinized, rehydrated, subjected to antigen‐retrieval treatment, and then blocked in 5% bovine serum albumin (BSA) + 5% normal goat serum in PBS for 1 h at room temperature. After overnight incubation with primary antibodies, including DPP4 (R&D Systems, Minneapolis, MN, USA, catalogue no. AF1180, 1:250), CD68 (Invitrogen, Waltham, MA, USA, catalogue no. PA5‐89134, 1:100), CD206 (Abcam, China, catalogue no. ab64693, 1:100), SEMA3C (Abclonal, China, catalogue no. A15386, 1:100), NRP‐1 (Santa, China, catalogue no. sc‐5307, 1:100), GAS6 (Santa, catalogue no. sc‐376087, 1:100), and AXL (Abclonal, catalogue no. A17874, 1:100) at 4 °C, the sections were washed three times with PBS and incubated with secondary antibodies for 1 h at room temperature. Sections were mounted using ProLong Gold Antifade Reagent with DAPI (Invitrogen, catalogue no. P36931), and fluorescence images were acquired using a Zeiss LSM 880 confocal microscope.

### Cell Isolation Using Fluorescence‐Activated Cell Sorting

The OAT SVFs from patients with TED (TED8–19, information provided in Table S1) and healthy controls (CON7‐12, information provided in Table S1) were pooled and resuspended in fluorescence‐activated cell sorting buffer and incubated for 45 min at 4 °C with fluorescein isothiocyanate (FITC) anti‐human CD31 antibody (BioLegend, San Diego, CA, USA, catalogue no. 303 104, 1:250), FITC anti‐human CD45 antibody (BioLegend, catalogue no. 368 508, 1:250), PE anti‐human PDGFRα antibody (BioLegend, catalogue no. 323 506, 1:100), APC anti‐human DPP4 antibody (BioLegend, catalogue no. 302 710, 1:100), and APC recombinant anti‐glypican 3 antibody (Sino Biological, China, catalogue no. 100393‐R024‐A, 1:100). DAPI (BioLegend, catalogue no. 422 801, 1:1000) was added for the final 15 min. Cells were washed three times with fluorescence activated cell sorting buffer to remove unbound antibodies, followed by cell sorting on a BD fluorescence activated cell sorting Aria cell sorter (BD Biosciences, San Jose, CA, USA) equipped with a 100‐µm nozzle, and the following lasers and filters: DAPI, 405 and 450/50 nm; FITC, 488 and 515/20 nm; PE, 532 and 780/60 nm, and APC, 640 and 780/60 nm. Instrument compensation for all fluorochromes was performed with SVF cells before each experiment. Once sorting was completed, we cultured DPP4^+^ and GPC3^+^ cells in DMEM/F12 supplemented with 10% FBS and 1% penicillin/streptomycin in a humidified incubator at 37 °C, 5% carbon dioxide. Isolated cells were used to confirm fibrosis and adipogenesis capacity using qRT‐PCR with corresponding indicators. Real‐time PCR primer sequences are listed in Table S2 (Supporting Information). GAPDH served as the reference gene for normalization, and relative expression changes were calculated using the 2^−△△Ct^ method.

### Induction of M1/M2 Macrophages

Macrophages, cultured in 1640 medium with 10% FBS (v/v), 100 IU mL^−1^ penicillin, and 50 µg mL^−1^ streptomycin sulphate, were induced from cell‐bank THP‐1 cells and monocytes extracted from peripheral blood samples of six patients with TED (TED20–25, information provided in Table S1, Supporting Information). We induced differentiation from THP‐1 cells to M0 macrophages by incubation with 100 ng mL^−1^ phorbol myristate acetate (PMA; Sigma, St. Louis, MO, USA, catalogue no.16561‐29‐8) for 48 h, and 50 ng mL^−1^ M‐CSF (Stemcell, Vancouver, Canada, catalogue no.78057) was added to the medium of monocytes for 8 days to induce M0 macrophage differentiation. Incubation with lipopolysaccharide (LPS; Sigma, catalogue no. SMB00610, 250 ng mL^−1^) for 48 h was used for M1 macrophage differentiation, and IL‐4 (Stemcell, catalogue no. 78 045, 20 ng mL^−1^) with IL‐13 (Stemcell, catalogue no. 78 029, 20 ng mL^−1^) was used for M2 macrophage polarization. We tracked the expression of specific marker genes (M1 markers: *TNFα*, *IL1B*, *IL6*, and *C‐C Motif chemokine receptor 7 (CCR7)*; M2 markers: *IL10*, *CCL18*, *CCL22*, *MRC1*, and *TGFB1*) to confirm M1/M2 induction, and *GAS6* expression in M0, M1, and M2 macrophages was quantified using qRT‐PCR. Real‐time PCR primer sequences are listed in Table S2 (Supporting Information). GAPDH served as the reference gene for normalization, and relative expression changes were calculated using 2^−△△Ct^ method.

### Cell–Cell Interaction Evaluation and Inhibition Assay

We evaluated cell–cell interactions between PDGFRα^+^DPP4^+^ cells, PDGFRα^+^GPC3^+^ cells, or SVF cells and M2 macrophages by incubating PDGFRα^+^DPP4^+^ cells, PDGFRα^+^GPC3^+^ cells, or SVF cells in M2 macrophage medium for 48 h. M0 macrophage medium was used as control. To eliminate interference from inducers in the cell–cell interaction experiment, the M2 and M0 macrophage medium was replenished for 48 h, and then added to PDGFRα^+^DPP4^+^, PDGFRα^+^GPC3^+^, or SVF cells (Figure 5a, and Figure S4, Supporting Information). We subsequently harvested PDGFRα^+^DPP4^+^, PDGFRα^+^GPC3^+^, and SVF cells for RNA extraction for qRT‐PCR and analyzed the supernatants of M0 and M2 macrophage cultures without inducers for GAS6 using an ELISA kit (Abclonal, catalogue no. RK01443) according to the manufacturer's instructions.

We performed inhibition assays by adding 125 nm TP0903 to PDGFRα^+^DPP4^+^ or PDGFRα^+^GPC3^+^ cell medium for 24 h or anti‐AXL siRNA to PDGFRα^+^DPP4^+^ cell medium for 48h, after which the medium was replaced with M2 macrophage supernatant or M0 macrophage supernatant (controls) for 48 h at 37 °C (Figure 7c and Figure S5d). We confirmed fibrosis using qRT‐PCR. Real‐time PCR primer sequences are listed in Table S2, Supporting Information. GAPDH served as the reference gene for normalization, and relative expression changes were calculated using the 2^−△△Ct^ method. Duplexed Silencer Select siRNA for AXL (Invitrogen, catalogue no. 4 390 824) was transfected into cells using Lipofectamine RNAiMAX (Invitrogen) in accordance with the manufacturer's instructions. Silencer Select siRNA for Negative Control no.1 (Invitrogen) was used as the scrambled control. Knockdown of AXL was confirmed by qRT‐PCR (Table S2, Supporting Information). GAPDH served as the reference gene for normalization, and relative expression changes were calculated using the 2^−△△Ct^ method.

### Luciferase‐mCherry Labeled OF‐CL for Tracing Transplantation

To co‐express luciferase and mCherry in OF‐CL, 293T cells were cultured in high‐glucose DMEM supplemented with 10% FBS and 1% penicillin‐streptomycin until they reached 80%–90% confluence. The plasmids used for transfection included the packaging plasmids (PMD2.G and PSPA×2), the core plasmids (Vector‐plvx‐pak‐puro, pCMV‐C‐mCherry [Beyotime, D2628], and pGL6‐CMV‐Luc [Bryotime, D2091]), and the transfection reagent Lipo8000. 24 h post‐transfection, the medium was replaced with fresh medium containing 2% FBS and 1% penicillin–streptomycin to support viral production. The viral supernatant was collected on days 2 and 3, filtered through a 0.22 µm filter, and stored at 4 °C for short‐term use or at −80 °C for long‐term storage.

The OF‐CL cells were seeded in a 6‐well plate and infected with the viral supernatant in the presence of 5 µg mL^−1^ polybrene to enhance infection efficiency. After 24 h, the infection medium was replaced with fresh medium, and the cells were cultured for an additional 24–48 h. To select for stably transduced cells, puromycin was added to the culture medium at a final concentration of 2 µg mL^−1^ for 48–72 h. The puromycin concentration was determined based on pre‐experimental titration of the resistance gene encoded by the plasmid. The selection process was continued for at least one week to ensure the survival of only the cells with stable transduction. The resulting OF‐CL cell line was verified for transgene expression and expanded for subsequent xenograft studies.

### Orthotopic Engraftment of TED Patient‐Derived Xenograft

Xenograft experiments were performed on 4–6‐week‐old NOD/SCID mice. All mice were purchased from GemPharmatech Laboratories and maintained with a standard 12‐h light‐dark cycle. The OF‐CL cells^[^ ^64^ ^]^ or primary SVF cells of patients with TED (TED26–28, information provided in Table S1, Supporting Information) were cultured in DMEM/F12 supplemented with 10% FBS. The cultures were treated for 48 h with or without 50 ng mL^−1^ GAS6, and with or without 125 nM TP0903. A total of 2×10^4^ cells were suspended in 10 µL Matrigel and introduced into the perioptic space with a 33‐gauge blunt‐end microinjection needle attached to a 10 µL syringe (Hamilton, Reno, NV, USA). Engrafted mice were inspected twice a week for intraocular changes. The animals were sacrificed after 2 weeks, and the orbital tissues were collected for H&E, Masson, and immunostaining with mCherry rat monoclonal antibody (Thermo/Life/Invitrogen, M11217) or anti‐Collagen I rabbit multiclonal antibody (Abcam, RM1131).

### Luciferase In Vivo Imaging

A working solution of D‐Luciferin potassium was prepared by dissolving 150 mg of D‐Luciferin potassium (MedChemExpress, HY‐12591B) in ultrapure water to a final concentration of 150 mg kg^−1^. Mice were anesthetized, and the prepared D‐Luciferin potassium solution was administered intraperitoneally at a dose of 100 µL per 10 g of body weight. The mice were allowed a period of 10–20 min following the injection to permit the light signal to reach its peak intensity before imaging. Subsequently, the anesthetized mice were placed in a prone position on the imaging platform of the in vivo imaging system (IVIS Spectrum CT, PerkinElmer). To ensure data stability and reliability, at least three replicate images were captured for each mouse.

### Magnetic Resonance Imaging

Magnetic Resonance Imaging (MRI) was performed using a magnetic resonance imaging system for small animals (Bruker BioSpec 94/20, USR) equipped with a Bruker BioSpin MRl GmbH (Rudolf‐Plank‐Strasse 23, D‐76275 Ettlingen/Germany) Animals were anesthetized with isoflurane and positioned on a dedicated mouse scan bed (Bruker BioSpin). Structural, high‐resolution images were acquired with a T2‐weighted TurboRARE sequence (TR/TEeff = 3500/31.76 ms, RARE factor = 8, NA = 4, spatial resolution = 0.098 mm x 0.098 mm x 0.5 mm, slices = 8, no gaps, scan time = 7 min 28 s). Axial T2‐weighted MR images were acquired to compare the proptosis of bilateral eyes, which was measured by the distance between the corneal apex and the ipsilateral orbit apex. A circular ROI (0.09 mm^2^) was placed within the extraocular muscle region (representing orbital fat), and another circular ROI with the same area was placed within the ipsilateral brain white matter. The tissue‐specific signal intensity ratio for each mouse (Signal Intensity _Orbital Fat/Brain_) was calculated as the ratio between the right eye (OD _Orbital Fat/Brain_) and the left eye (OS _Orbital Fat/Brain_).^[^ ^65^ ^]^

### Statistical Analysis

We performed gene ontology enrichment analysis in scRNA‐seq using the “enrichGO” function, and p‐values were calculated using a hypergeometric test and adjusted for multiple hypothesis testing with the Benjamini–Hochberg procedure. Experiments were non‐randomized and non‐blinded. We used the unpaired *t*‐test for two‐group differential analysis. Statistical analyses were conducted using IBM SPSS Statistics (version 21.0) and GraphPad Prism Software (version 8.4.0). Statistical significance was defined as p < 0.05, and all experimental results are given as mean ± standard deviation (SD) with at least three repeats.

## Conflict of Interest

The authors declare no conflict of interest.

## Supporting information



Supporting Information

## Data Availability

Raw sequence data from scRNA‐seq analysis reported in this paper have been deposited (PRJCA015904) in the Genome Sequence Archive in the BIG Data Center, Chinese Academy of Sciences under accession codes HRA005900.
