# Supplementary material for: PDGFRα+DPP4+ Fibroblasts‐Macrophage Crosstalk Induces Orbital Fibrosis in Treatment‐Resistant Thyroid Eye Disease via the GAS6‐AXL Pathway
Source: Adv Sci (Weinh). 2025 Sep 29;12(45):e11404. doi: 10.1002/advs.202511404 (PMC12677676; doi:10.1002/advs.202511404)
Supplement: Supplementary file 1 — Supporting Information [file ADVS-12-e11404-s001.docx]

Supporting Information

**PDGFRα+DPP4+ fibroblasts-macrophage crosstalk induces orbital fibrosis**

**in treatment-resistant thyroid eye disease via the GAS6-AXL pathway**

Lu Cheng, Jinwei Cheng, Guiling Liang, Xiaorui Wang, Yanwen Ge, Jiapei Liu, Luwei Cai, Hui Ying, Fenfen Wang, Ning Shen, Jing Hu, Yufan Wang, Philipp E. Scherer, Ben Zhou,* Mengle Shao,* Xiaodong Sun,* Fang Zhang*

**Table S1.** Clinical characteristics of all Thyroid eye disease (TED) patients in this study

| Patient | Gender | Age | Smoking  (months) | TED duration (months) | Proptosis (mm) | | Clinical activity score | | Severity | Therapy history | Thyroid function |
| --- | --- | --- | --- | --- | --- | --- | --- | --- | --- | --- | --- |
|  |  |  |  |  | OD | OS | OD | OS |  |  |  |
| TED 1 | Male | 18 | No | 5 | 24 | 24 | 1/7 | 1/7 | Moderate-to-severe | ST | E |
| TED 2 | Female | 27 | No | 24 | 20.5 | 22 | 0/7 | 0/7 | Moderate-to-severe | ATT | E |
| TED 3 | Female | 53 | No | 24 | 21 | 23 | 1/7 | 1/7 | Moderate-to-severe | ST, SIT | E |
| TED 4 | Female | 35 | No | 36 | 23 | 22 | 2/7 | 2/7 | Moderate-to-severe | SIT | SHO |
| TED 5 | Male | 49 | No | 36 | 19 | 19 | 1/7 | 1/7 | Moderate-to-severe | ATT, ST | SHO |
| TED 6 | Male | 59 | No | 12 | 17 | 17 | 0/7 | 0/7 | Moderate-to-severe | ATT, ST | E |
| TED 7 | Male | 31 | No | 96 | 22 | 22 | 0/7 | 0/7 | Moderate-to-severe | ATT | E |
| TED 8 | Male | 59 | No | 7 | 21.5 | 19 | 1/7 | 0/7 | Moderate-to-severe | ST | E |
| TED 9 | Female | 29 | No | 14 | 18 | 20 | 0/7 | 0/7 | Moderate-to-severe | ATT, ST, SIT | E |
| TED 10 | Male | 78 | No | ≥ 240 | 22 | 25 | 1/7 | 1/7 | Moderate-to-severe | ATT | E |
| TED 11 | Male | 26 | No | 34 | 20 | 20 | 1/7 | 1/7 | Moderate-to-severe | ATT, ST | E |
| TED 12 | Male | 20 | No | 30 | 21 | 21 | 1/7 | 1/7 | Moderate-to-severe | ATT, ST, SIT | SHO |
| TED 13 | Female | 35 | No | 36 | 22 | 23 | 2/7 | 2/7 | Moderate-to-severe | ATT, ST | E |
| TED 14 | Female | 54 | No | 6 | 19 | 22 | 2/7 | 1/7 | Moderate-to-severe | ATT | E |
| TED 15 | Female | 51 | No | 12 | 19 | 22 | 2/7 | 1/7 | Moderate-to-severe | ATT, ST | SHE |
| TED 16 | Male | 27 | No | 6 | 21 | 18 | 0/7 | 0/7 | Moderate-to-severe | ATT | E |
| TED 17 | Female | 33 | No | 30 | 14 | 17 | 1/7 | 1/7 | Moderate-to-severe | ST | E |
| TED 18 | Male | 19 | No | 24 | 19 | 19 | 2/7 | 2/7 | Moderate-to-severe | SIT | SHO |
| TED 19 | Female | 31 | No | 32 | 19 | 18 | 2/7 | 2/7 | Moderate-to-severe | ST, SIT | E |
| TED 20 | Female | 45 | No | 12 | 11 | 10 | 0/7 | 0/7 | Moderate-to-severe | SIT | E |
| TED 21 | Female | 24 | No | 56 | 21 | 20 | 0/7 | 1/7 | Moderate-to-severe | ATT | SHE |
| TED 22 | Male | 56 | 360 | 12 | 12 | 11 | 0/7 | 2/7 | Moderate-to-severe | ATT, ST | SHE |
| TED 23 | Female | 47 | No | 36 | 16 | 20 | 2/7 | 2/7 | Moderate-to-severe | ATT, ST, SIT | SHO |
| TED 24 | Female | 26 | No | 28 | 18 | 21 | 1/7 | 1/7 | Moderate-to-severe | ATT, ST | E |
| TED 25 | Female | 43 | No | 60 | 22 | 23 | 1/7 | 1/7 | Moderate-to-severe | ATT, ST | E |
| TED 26 | Female | 45 | No | 36 | 22 | 22 | 0/7 | 0/7 | Moderate-to-severe | ATT, ST, SIT | SHO |
| TED 27 | Female | 26 | No | 24 | 17 | 19 | 0/7 | 0/7 | Moderate-to-severe | ATT | E |
| TED 28 | Male | 38 | No | 53 | 21 | 21 | 2/7 | 2/7 | Moderate-to-severe | ATT, ST | E |
| CON1 | Female | 66 | No | / | / | / | / | / | / | / | / |
| CON2 | Male | 60 | No | / | / | / | / | / | / | / | / |
| CON3 | Male | 68 | No | / | / | / | / | / | / | / | / |
| CON4 | Female | 67 | No | / | / | / | / | / | / | / | / |
| CON5 | Female | 67 | No | / | / | / | / | / | / | / | / |
| CON6 | Female | 32 | No | / | / | / | / | / | / | / | / |
| CON7 | Male | 26 | No | / | / | / | / | / | / | / | / |
| CON8 | Female | 42 | No | / | / | / | / | / | / | / | / |
| CON9 | Female | 55 | No | / | / | / | / | / | / | / | / |
| CON10 | Female | 30 | No | / | / | / | / | / | / | / | / |
| CON11 | Female | 60 | No | / | / | / | / | / | / | / | / |
| CON12 | Male | 21 | No | / | / | / | / | / | / | / | / |

OD, Oculus Dexter; OS, Oculus Sinister; SIT, Radioactive iodine treatment; ST, Steroid treatment; ATT, Anti-thyroid treatment; SHO, subclinical hypothyroidism; SHE, subclinical hyperthyroidism; E, Euthyroid.

TED1–3 and CON1–2, scRNA-seq; TED3–7 and CON3–9, H&E, Masson and immunofluorescence; TED8–19 and CON7-12, flow cytometry and *in vitro* experiments; TED20–25, human monocyte isolation and *in vitro* experiments; TED26-28, xenografts of *in vivo* experiments

**Table S2.** Real-time PCR primer sequences

| Primer | Sequences (5' to 3') |
| --- | --- |
|  |  |
| *COL1A1*-Forward | GATTCCCTGGACCTAAAGGTGC |
| *COL1A1*-Reverse | AGCCTCTCCATCTTTGCCAGCA |
| *COL3A1*-Forward | CACTCAAAAGCAGCGTGGAC |
| *COL3A1*-Reverse | GGCCACCGAGAAGACTTTGA |
| *ELN*-Forward | GGTTGTGTCACCAGAAGCAGCT |
| *ELN*-Reverse | CCGTAAGTAGGAATGCCTCCAAC |
| *FN1*-Forward | ACAACACCGAGGTGACTGAGAC |
| *FN1*-Reverse | GGACACAACGATGCTTCCTGAG |
| *ACTA2*-Forward | CTATGCCTCTGGACGCACAACT |
| *ACTA2*-Reverse | CAGATCCAGACGCATGATGGCA |
| *TIMP1*-Forward | GGAGAGTGTCTGCGGATACTTC |
| *TIMP1*-Reverse | GCAGGTAGTGATGTGCAAGAGTC |
| *PPARγ*-Forward | AGCCTGCGAAAGCCTTTTGGTG |
| *PPARγ*-Reverse | GGCTTCACATTCAGCAAACCTGG |
| *CEBPα*-Forward | AGGAGGATGAAGCCAAGCAGCT |
| *CEBPα*-Reverse | AGTGCGCGATCTGGAACTGCAG |
| *CD36*-Forward | AAGCCAGGTATTGCAGTTCTTT |
| *CD36*-Reverse  *GAS6*-Forward  *GAS6*-Reverse  *AXL*-Forward  *AXL*-Reverse | GCATTTGCTGATGTCTAGCACA  CTCGTGCAGCCTATAAACCCT  TCCTCGTGTTCACTTTCACCG  ATCAGCTTCGGCTAGGCAG  TCCGCGTAGCACTAATGTTCT |
| *GAPDH-*Forward | AGCCTCAAGATCATCAGCAATGCC |
| *GAPDH-*Reverse | TGTGGTCATGAGTCCTTCCACGAT |


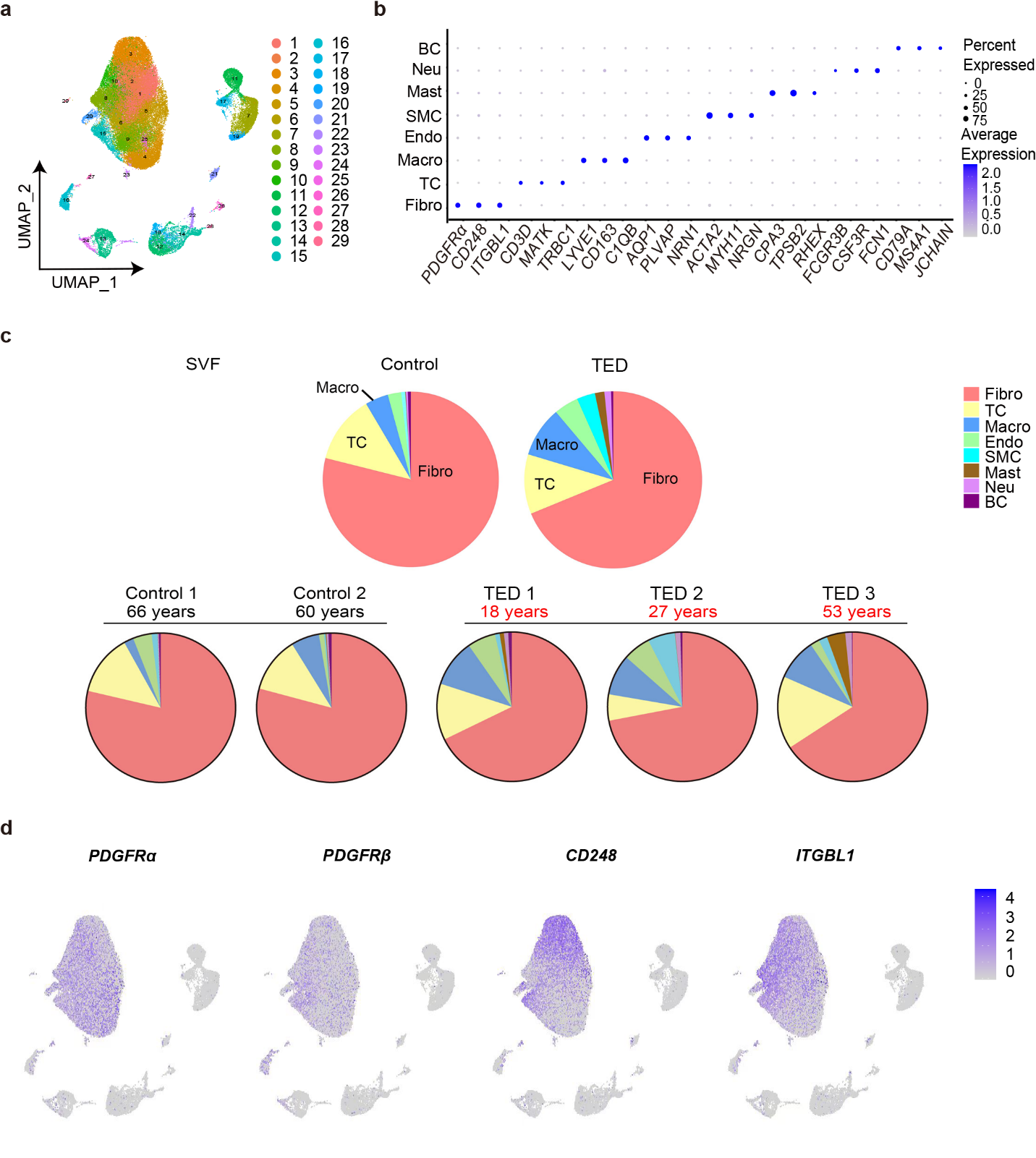


**Figure. S1. Clustering illustration of scRNA-seq in orbital adipose tissues.** (**a**) Clustering results for stromal vascular fraction (SVF) samples isolated from orbital adipose tissues (OAT) of three patients with Thyroid eye disease (TED) and two control subjects. Samples were assigned to 29 distinct clusters. (**b**) Dot plot showing classical marker gene expressions in each cell cluster. Dot size indicates the proportion of cells expressing the denoted gene, and colour represents the average normalized expression level within the cluster. (**c**) Pie diagram showing the relative proportion of major cell types within human SVF derived from OAT samples of two control and three Thyroid eye disease (TED) patients respectively. (**d**) Feature plot indicating the distribution and expression of representative fibrosis markers in all the SVF cells. Each dot represents a single cell.

Fibro, fibroblasts; TC, T cells; Macro, macrophages; Endo, endothelial cells; SMC, smooth muscle cells; Mast, mast cells; Neu, neutrophils; BC, B cells.


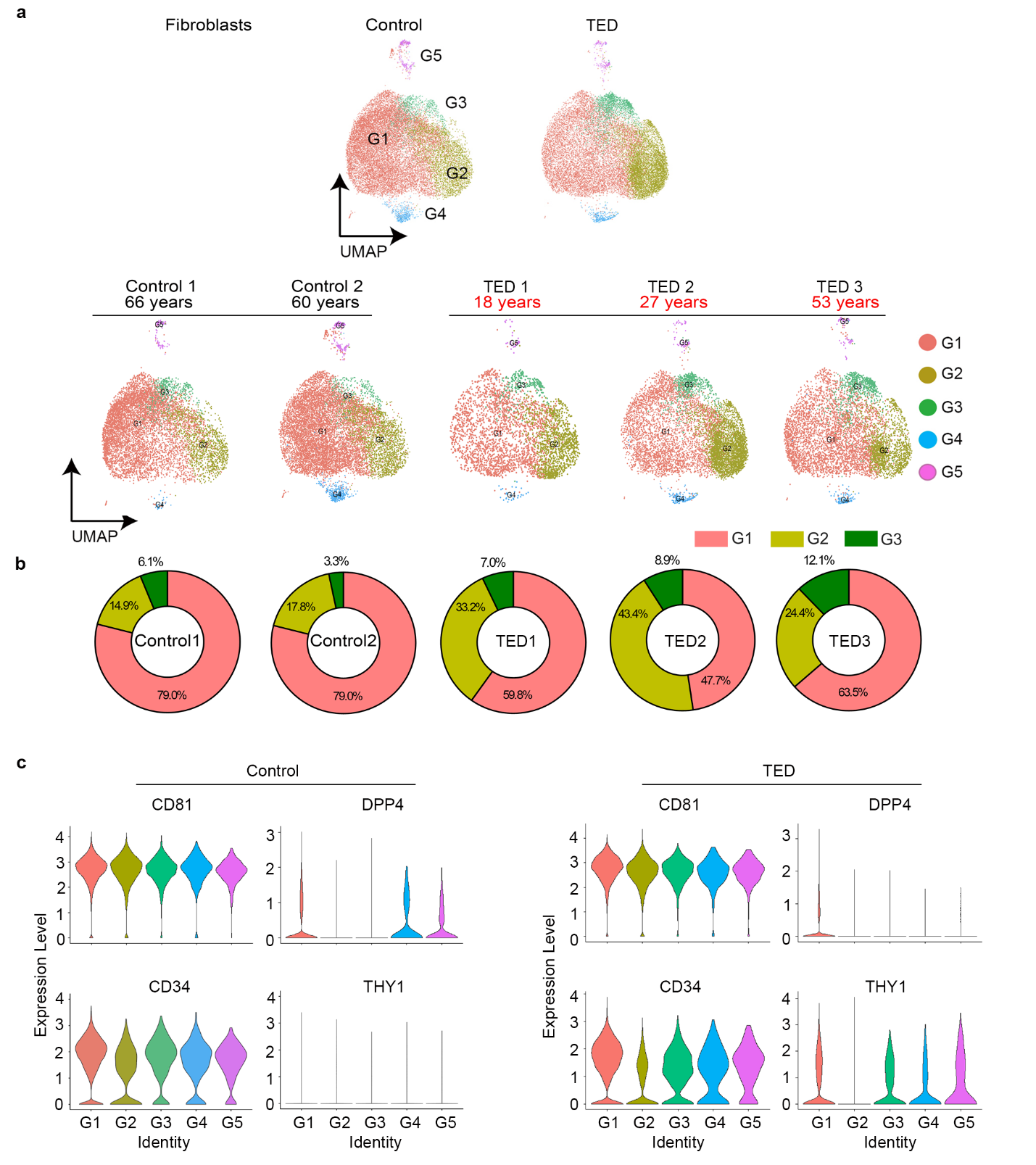


**Figure. S2. Additional clustering results and pseudotime cell ordering of fibroblasts in three TED samples.** (**a**) Two-dimensional UMAP visualization of orbital fibroblast subpopulations from SVF of two control and three TED patients. (**b**) The pie diagram illustrates the relative proportions of the three major subgroups (G1, G2 and G3) in orbital fibroblasts from each sample in (a). (**c**) Violin plot showing the expression patterns of published progenitor-associated marker genes, including *CD81, DPP4, THY1,* and *CD34*, within distinct fibroblast subgroups derived from control subjects and TED patients.


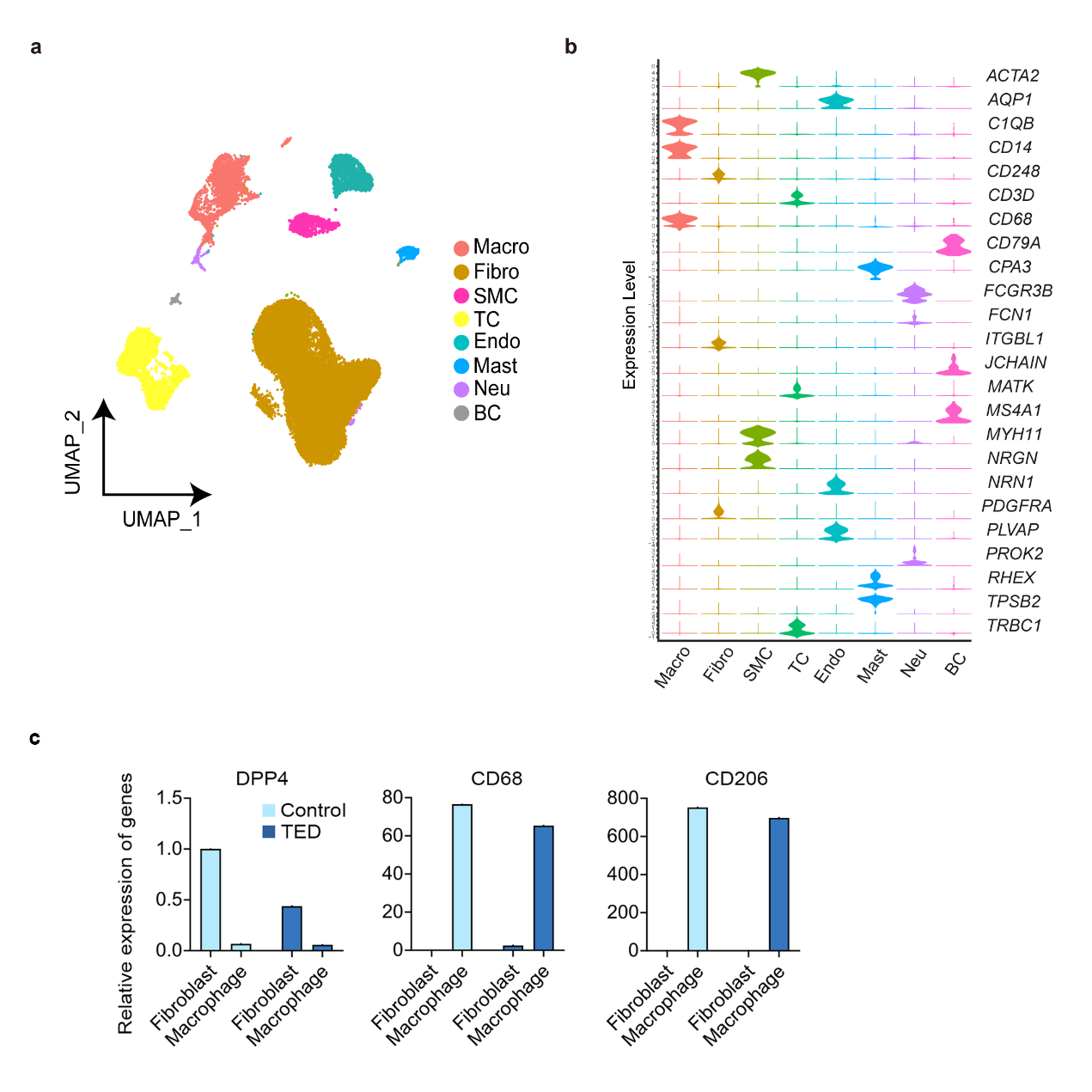


**Figure. S3. Comparison of marker genes in the stromal vascular fraction (SVF) between TED patients and control patients.** (**a**) Re-clustering results of SVF cells from three TED samples for further cell–cell communication analysis. (**b**) Violin plot of classical marker genes in each cell group from three TED samples. (**c**) Distinct expression patterns of DPP4, CD68, and CD206 genes in orbital fibroblasts and macrophages derived from the SVF of control and TED samples.


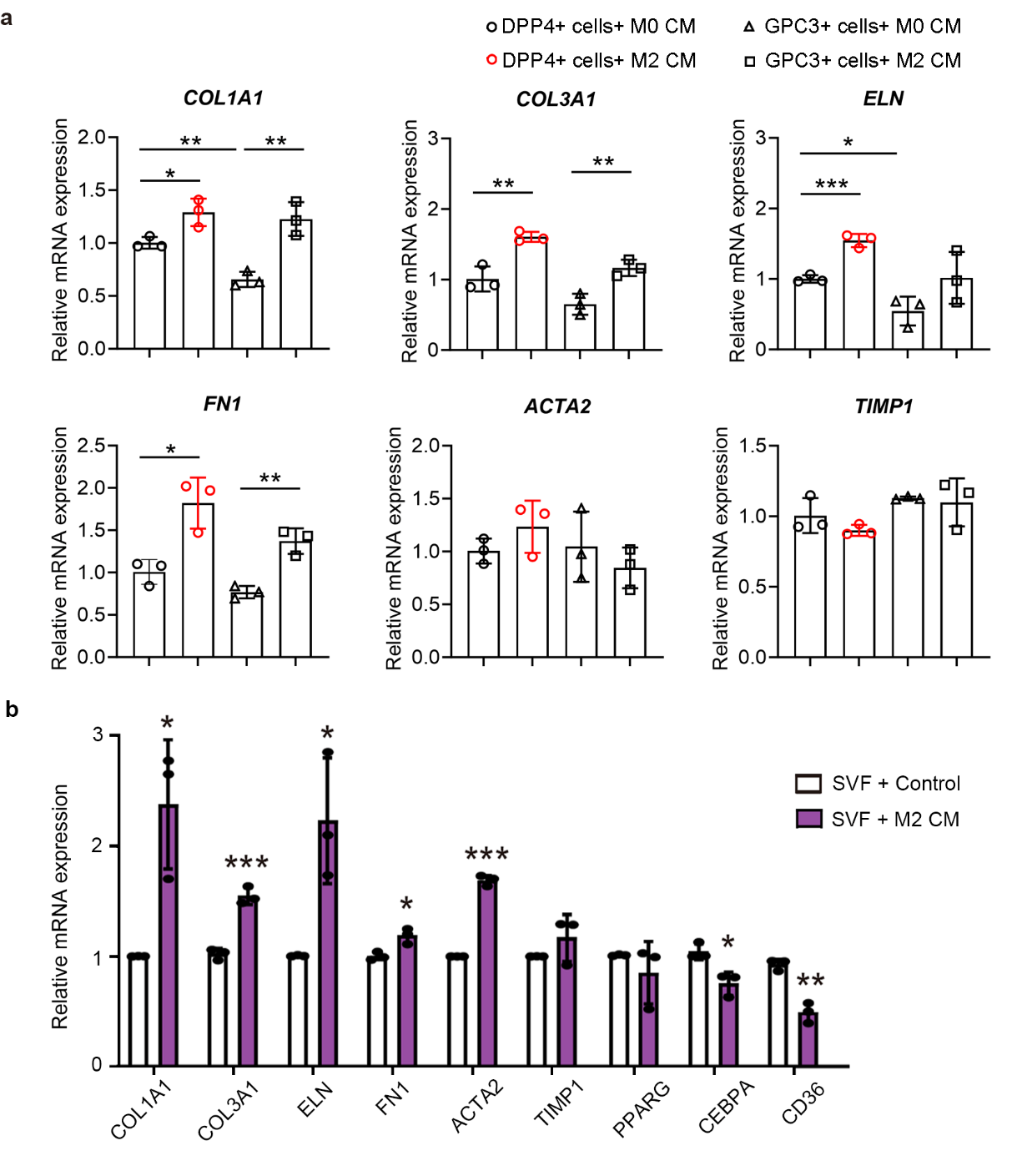


**Figure. S4. M2 macrophage-derived conditioned medium activates both PDGFRα^+^DPP4^+^ fibroblasts and SVF derived from human OAT, promoting fibrosis in vitro.** (**a)** qRT-PCR analysis results of fibrosis-associated gene expression in PDGFRα^+^DPP4^+^ fibroblasts (labeled DPP4^+^ in the figure) and PDGFRα^+^GPC3^+^ cells (labeled GPC3^+^ in the figure) from control subjects following 48-hour treatment with M2 or M0 macrophages supernatant. (**b**) qRT-PCR analysis results of fibrosis- and adipogenesis-associated gene expression of SVF cells from TED patients after treatment with the supernatant of M2 or M0 macrophages for 48 hours. M0 macrophage-conditioned medium was used as control. Data are presented as mean values ± standard deviation (SD) (n = 3 biologically independent experiments). Two-sided unpaired *t*-test, *p < 0.05, **p < 0.01, ***p < 0.001.


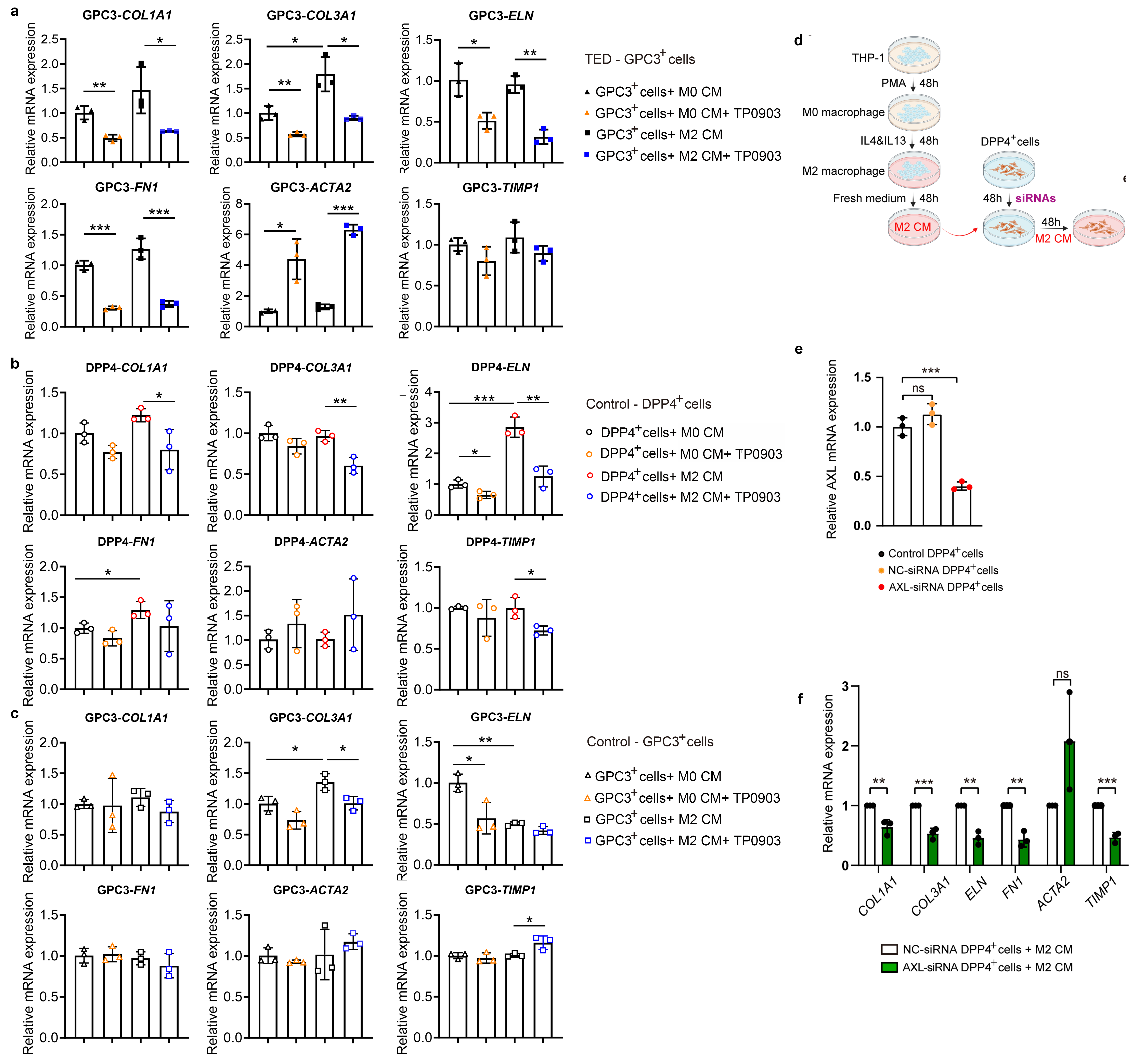


**Figure. S5. Both the AXL inhibitor TP0903 and siRNA inhibit fibrosis in PDGFRα^+^DPP4^+^ fibroblasts induced by THP-1 derived M2 macrophages.** (**a-c**) qRT-PCR analysis of mRNA expression of fibrosis-associated gene mRNA expression in 3 control groups of PDGFRα^+^DPP4^+^ fibroblasts: (**a**) PDGFRα^+^GPC3^+^ cells (labeled GPC3^+^ in the figure) from TED patients, and (**b**) PDGFRα^+^DPP4^+^ (labeled DPP4^+^ in the figure) and (**c**) PDGFRα^+^GPC3^+^ cells (labeled GPC3^+^ in the figure) from control subjects, following treatment with M2 or M0 macrophage-conditioned medium, in the present and absent of TP0903. M0 macrophage-conditioned medium was used as control. Data are presented as mean ± SD (n = 3, representing three biologically independent experiments conducted using three different patient-derived cells). Two-sided unpaired *t*-test, *p < 0.05, **p < 0.01, ***p < 0.001. (**d**) Schematic representation of *in vitro* experimental investigation of AXL knockdown PDGFRα^+^DPP4^+^ cells and THP-1-induced M2 macrophage interactions. (**e**) Comparison of relative AXL mRNA expression in control, negative-control siRNA (NC-siRNA) and AXL-siRNA treated PDGFRα^+^DPP4^+^ cells. Data are presented as mean ± standard deviation (SD). Two-sided unpaired t-test, ***p < 0.001, ns, no significance. (**f**) qRT-PCR analysis results of fibrosis-associated gene expression of control and AXL knockdown PDGFRα^+^DPP4^+^ fibroblasts after treatment with the supernatant of M2 macrophages. Data are presented as mean ± SD (n = 3 biologically independent experiments). Two-sided unpaired t-test, ns indicates no significance, **p < 0.01, ***p < 0.001.


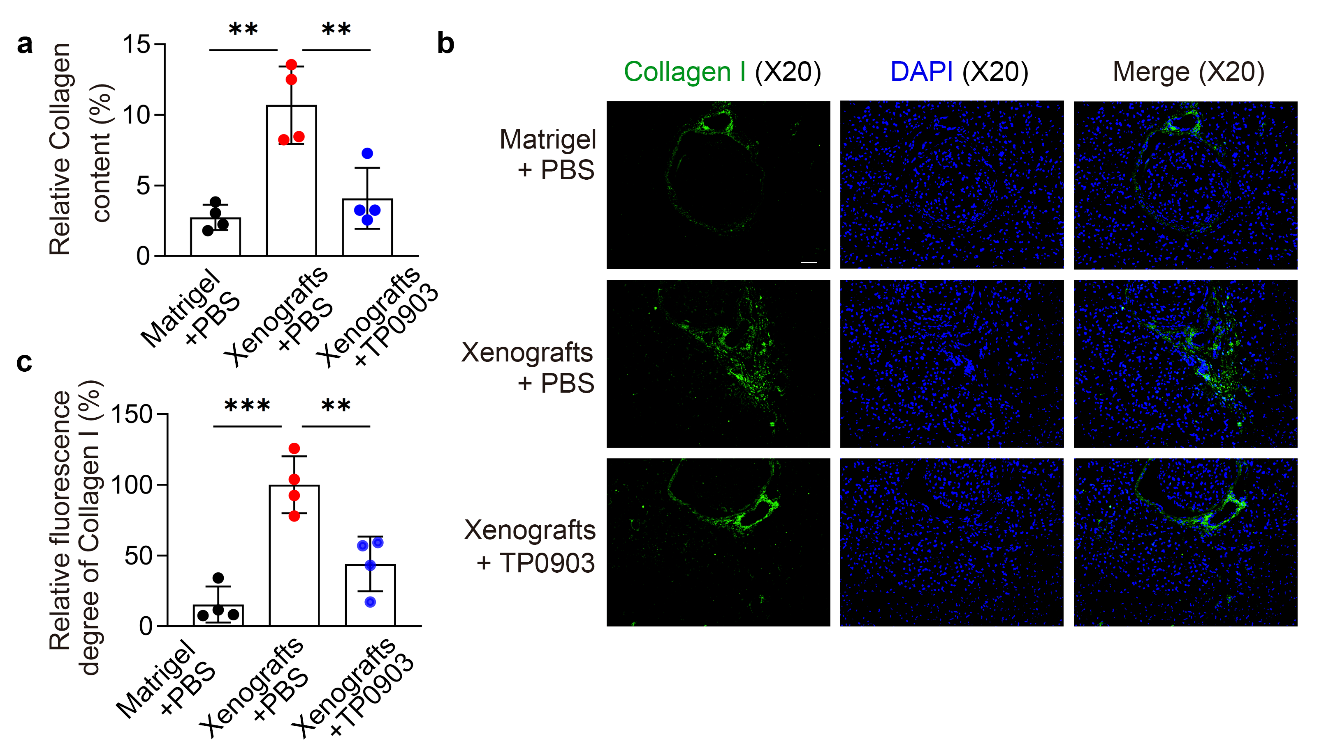


**Figure. S6. Histopathological analyses indicate that TP0903 suppresses orbital adipose fibrosis in vivo.** (a) Quantification of relative collagen content by Masson’s trichrome staining of orbital tissue sections from orthotopic xenograft models injected with TED patient-derived orbital fibroblasts. Columns represent: control group (Matrigel + PBS), xenograft group (Xenografts + PBS), and TP0903-pretreated xenograft group (Xenografts + TP0903). Data are presented as mean ± SD (n = 4).  Two-sided unpaired *t-*test, **p < 0.01. (**b**) Representative immunofluorescence images of Collagen I (with DAPI nuclear counterstain) from orbital tissue sections at the injection site (corresponding to Figure 8g). Rows correspond to the following groups: control group (Matrigel + PBS), xenografts group (Xenografts + PBS), and xenografts pretreated with TP0903 group (Xenografts + TP0903). Scale bar = 50 μm. (c) Statistical analysis of relative fluorescence degree of Collagen I using immunofluorescence staining. Data are presented as mean ± SD (n = 4). Two-sided unpaired *t-*test, **p < 0.01, ***p < 0.001.


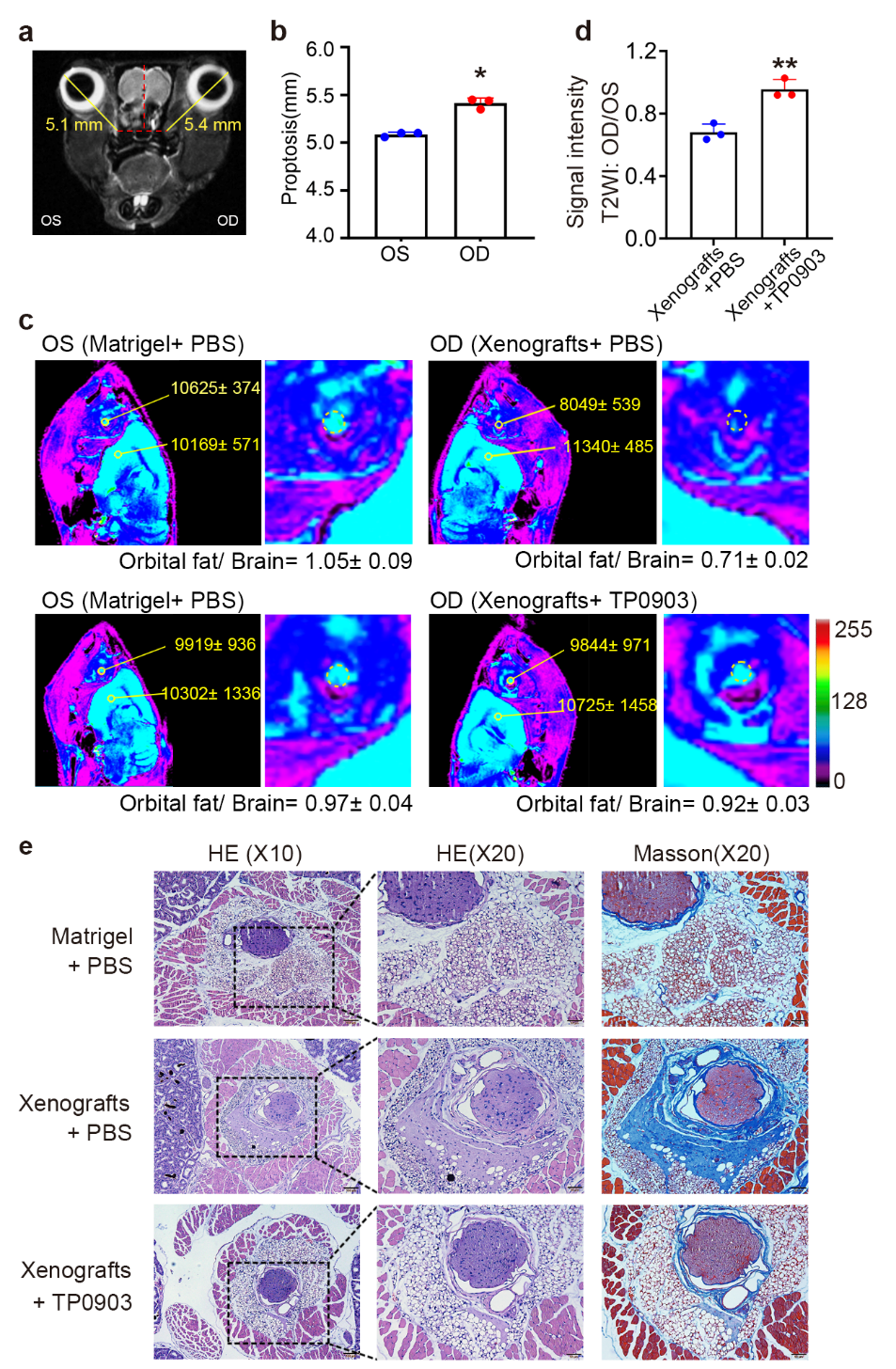


**Figure. S7. TP0903 inhibits orbital adipose fibrosis in orthotopic models with** **TED patient-derived SVF cells.** (**a**) Axial magnetic resonance imaging (MRI) images showed an increased in proptosis of the experimental right eye compared with the control left eye. Orthotopic xenografts in the right compartment (oculus dexter, OD) mouse orbits were established by injection TED patient-derived SVF cells. In contrast, the left orbital compartment (oculus sinister, OS) mouse orbits were injected with Matrigel as control. (**b**) Statistical analysis of proptosis in bilateral eyes following intra-orbital injections. Data are presented as mean ± standard deviation (SD) (n = 3). Two-sided paired *t-*test, *p < 0.05. (**c**) Typical pseudo-color MRI of orthotopic mouse models obtained by injection of TED patient-derived SVF cells (n = 3 patients). The signal intensity (Orbital Fat/ Brain) of left eyes injected with Matrigel (left panel) and right eyes injected with SVF cells (right panel) with or without TP0903 treatment are shown. (d) Statistical analysis of the OD/OS signal intensity (Orbital Fat/ Brain) between the xenograft models with or without TP0903 treatment. Data are presented as mean ± SD (n = 3 patients). Two-sided unpaired t-test, **p < 0.01. (**e**) Typical images of continuous section H&E and Masson staining in the control group (Matrigel + PBS), orthotopic group (Xenografts + PBS), and TP0903-pretreated orthotopic group (Xenografts + TP0903). Each row represents the injection area of intra-orbital tissues with H&E staining magnified at ×100, ×200 and Masson staining magnified at ×200, respectively (n = 3).
